# Feeding rates of malaria vectors from a prototype attractive sugar bait station in Western Province, Zambia: results of an entomological validation study

**DOI:** 10.1186/s12936-023-04491-9

**Published:** 2023-03-01

**Authors:** Javan Chanda, Joseph Wagman, Benjamin Chanda, Tresford Kaniki, Mirabelle Ng’andu, Rayford Muyabe, Mwansa Mwenya, Jimmy Sakala, John Miller, Gift Mwaanga, Limonty Simubali, Monicah Mirai Mburu, Edgar Simulundu, Alice Mungo, Keith Fraser, Lazaro Mwandigha, Ruth Ashton, Joshua Yukich, Angela F. Harris, Thomas R. Burkot, Erica Orange, Megan Littrell, Julian Entwistle

**Affiliations:** 1 PATH, Lusaka, Zambia; 2 PATH, Washington, DC USA; 3 Present Address: Jhpeigo, Lusaka, Zambia; 4 Macha Research Trust, Choma, Zambia; 5 Imperial College London, London, UK; 6 Present Address: University of Oxford, Oxford, UK; 7 School of Public Health and Tropical Medicine, Tulane University, New Orleans, USA; 8 IVCC, Liverpool, UK; 9 Australian Institute of Tropical Health and Medicine, James Cook University, Cairns, Australia; 10 PATH, Seattle, USA

## Abstract

**Background:**

Attractive targeted sugar bait (ATSB) stations are a promising new approach to malaria vector control that could compliment current tools by exploiting the natural sugar feeding behaviors of mosquitoes. Recent proof of concept work with a prototype ATSB^®^ Sarabi Bait Station (Westham Co., Hod-Hasharon, Israel) has demonstrated high feeding rates and significant reductions in vector density, human biting rate, and overall entomological inoculation rate for *Anopheles gambiae *sensu lato (*s.l.)* in the tropical savannah of western Mali. The study reported here was conducted in the more temperate, rainier region of Western Province, Zambia and was designed to confirm the primary vector species in region and to estimate corresponding rates of feeding from prototype attractive sugar bait (ASB) Sarabi Bait Stations.

**Methods:**

The product evaluated was the Sarabi v1.1.1 ASB station, which did not include insecticide but did include 0.8% uranine as a dye allowing for the detection, using UV fluorescence light microscopy, of mosquitoes that have acquired a sugar meal from the ASB. A two-phase, crossover study design was conducted in 10 village-based clusters in Western Province, Zambia. One study arm initially received 2 ASB stations per eligible structure while the other initially received 3. Primary mosquito sampling occurred via indoor and outdoor CDC Miniature UV Light Trap collection from March 01 through April 09, 2021 (Phase 1) and from April 19 to May 28, 2021 (Phase 2).

**Results:**

The dominant vector in the study area is *Anopheles funestus s.l.,* which was the most abundant species group collected (31% of all Anophelines; 45,038/144,5550), had the highest sporozoite rate (3.16%; 66 positives out of 2,090 tested), and accounted for 94.3% (66/70) of all sporozoite positive specimens. Of those *An. funestus* specimens further identified to species, 97.2% (2,090/2,150) were *An. funestus *sensu stricto (*s.s*.). *Anopheles gambiae s.l.* (96.8% of which were *Anopheles arabiensis*) is a likely secondary vector and *Anopheles squamosus* may play a minor role in transmission. Overall, 21.6% (9,218/42,587) of *An. funestus* specimens and 10.4% (201/1,940) of *An. gambiae* specimens collected were positive for uranine, translating into an estimated daily feeding rate of 8.9% [7.7–9.9%] for *An. funestus* (inter-cluster range of 5.5% to 12.7%) and 3.9% [3.3–4.7%] for *An. gambiae* (inter-cluster range of 1.0–5.2%). Feeding rates were no different among mosquitoes collected indoors or outdoors, or among mosquitoes from clusters with 2 or 3 ASBs per eligible structure. Similarly, there were no correlations observed between feeding rates and the average number of ASB stations per hectare or with weekly rainfall amounts.

**Conclusions:**

*Anopheles funestus* and *An. gambiae* vector populations in Western Province, Zambia readily fed from the prototype Sarabi v1.1.1 ASB sugar bait station. Observed feeding rates are in line with those thought to be required for ATSB stations to achieve reductions in malaria transmission when used in combination with conventional control methods (IRS or LLIN). These results supported the decision to implement a large-scale, epidemiological cluster randomized controlled trial of ATSB in Zambia, deploying 2 ATSB stations per eligible structure.

**Supplementary Information:**

The online version contains supplementary material available at 10.1186/s12936-023-04491-9.

## Background

Vector control has contributed substantially to the global reduction in malaria burden that has been observed since 2000 [1]. In sub-Saharan Africa, between 2000 and 2015, expanded access to the two vector control interventions recommended broadly by the World Health Organization (WHO) Global Malaria Programme (GMP), namely insecticide-treated bed nets (ITNs) and indoor residual spraying (IRS), is estimated to have averted more than 515,000,000 cases of malaria—more than 80% of the total progress in malaria case reduction made across the region during that time [2]. Furthermore, vector control remains one of the pillars of the Global Technical Strategy for Malaria 2016–2030 (GTS) [3] and is widely recognized as an essential component of malaria control and elimination programmes worldwide [4, 5].

However, the sustained efficacy of currently available tools is threatened. Key challenges include insecticide resistance, which is expanding and intensifying in vector populations across Africa [6–10], and the reality that sustained widespread use of IRS and ITNs, while key to maintaining the progress that has been achieved, may provide limited additional impact in many settings because these interventions don’t effectively target transmission by mosquitoes that do not (1) bite indoors at night when people are using a bed net, or (2) rest indoors on treated surfaces [11, 12]. These challenges are especially worrisome considering the current plateau in progress towards global malaria burden reduction goals, and the realization that many of the key targets of the GMP technical strategy are likely to be missed [1, 4, 5, 13]. Accordingly, the need to develop and quickly scale up new malaria vector control tools and innovative strategies to target residual transmission has been identified as a top global public health priority [1, 11, 13].

One promising new approach to malaria vector control is the use of attractive targeted sugar baits (ATSBs), which compliment current tools by exploiting the natural sugar feeding behaviors of mosquitoes [14–17]. In contrast to the blood-feeding behaviors exploited by ITNs and the resting behaviours exploited by IRS [18, 19], ATSBs use a combination of an attractive sugar-based bait and an oral toxin in an attract-and-kill approach that targets a novel part of the mosquito life cycle [18, 19].

The ATSB^®^ Sarabi Bait Station (Westham Co., Hod-Hasharon, Israel) was designed to attract and kill malaria vectors. Early proof of concept work guiding product development was completed in Mali, including a large-scale entomological field trial of early prototype Sarabi Bait Stations against *Anopheles gambiae *sensu lato (*s.l.)* in the tropical savannah of Kayes Region that demonstrated high ATSB feeding rates and significant reductions in *An. gambiae* density, biting rates, and overall entomological inoculation rate (EIR) [14]. Following these favorable results in Mali, the ATSB research team in Zambia, working in collaboration with the National Malaria Elimination Programme (NMEP) under the Zambian Ministry of Health (MoH), was engaged to conduct a series of entomological studies to investigate the potential for the Sarabi Bait Station to control malaria vectors at study sites in the more temperate, rainier region of Western Province, Zambia. Other key differences between this Zambian setting and the region in Mali where the ATSB proof of concept work was conducted include a greater vector species diversity, more abundant natural sugar sources, and more dispersed housing.

**Fig. 1:**
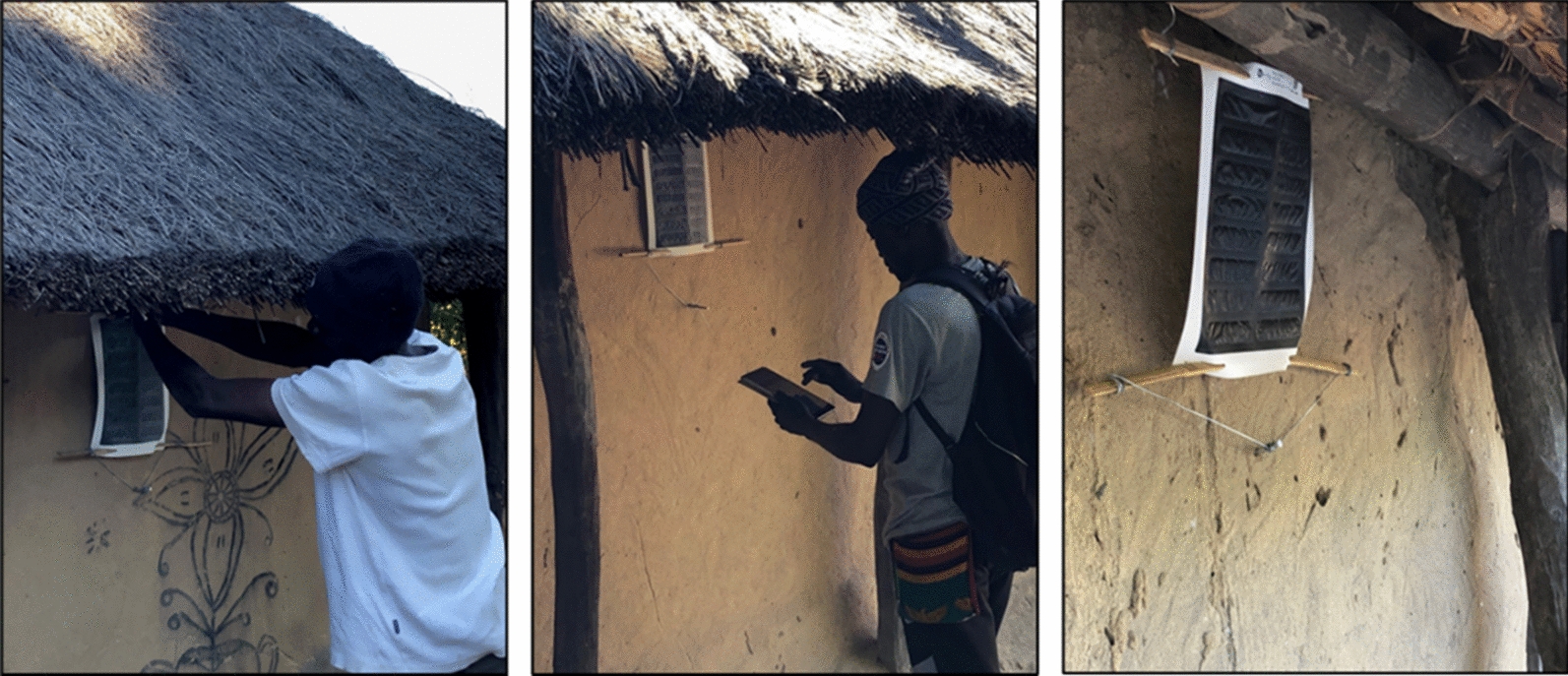
ASB station installation and final positioning during the feeding validation study

Following a series of cage and semi-field studies in Zambia, the ATSB research team designed the present entomological validation study to confirm the primary vector species in the study areas and to estimate their rates of sugar feeding from prototype attractive sugar bait (ASB) Sarabi v1.1.1 Bait Stations (Fig. 1). These prototype ASB stations contained no toxicant but did contain the fluorescent dye uranine as a way to assess ASB feeding in individual mosquitoes sampled from the local vector population [20]. The primary objective of the study was to estimate the daily ASB feeding rate for the dominant malaria vector(s) when either two or three ASB stations were installed on eligible structures throughout the community. The goal in generating this data was to inform implementation of a large-scale randomized control trial (RCT) of the Sarabi ATSB in Western Province.

The subsequent RCT would be designed to evaluate the impact of the ATSB on malaria transmission with a target of achieving a 30 percent reduction in malaria incidence over two transmission seasons. Adaptation to the conditions in Western Province, Zambia, of a mathematical model of ATSB performance in Mali [21] suggested that this target reduction in malaria incidence should be achieved with an incremental daily adult female vector mortality rate of approximately 2.5 percent, which in this study was estimating by calculating the daily feeding rate on ASB stations of vectors sampled in and around households where ASB stations had been installed. As such, this validation study was designed to measure if this target feeding rate of 2.5 percent nightly could be achieved with installation of two and/or three ASB stations per eligible structure. Results would be used to determine whether or not the full-scale ATSB RCT would proceed in this context (i.e., if the target feeding rate is achievable in Western Zambia) and to inform the number of bait stations per structure required to achieve the desired impact.

## Methods

### Study site

**Fig. 2:**
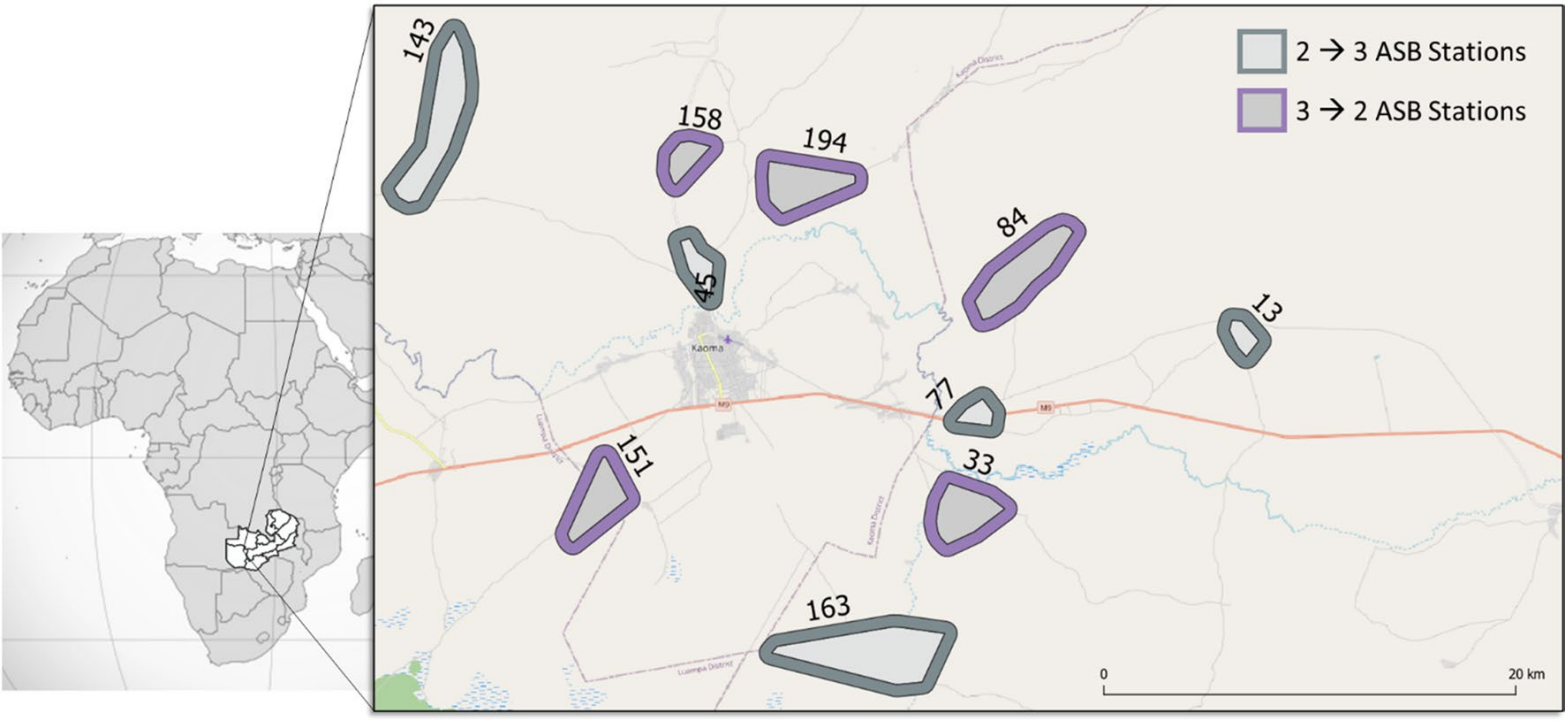
The ten clusters enrolled in the ASB feeding validation crossover study, located within the districts of Kaoma (northwestern clusters) and Nkeyema (southeastern clusters)

The study was conducted in 10 village-based clusters in Western Province, Zambia within Nkeyema and Kaoma districts (Fig. 2). Malaria transmission in Western Province is seasonal, typically characterized by peak transmission from January to May, corresponding to the annual rainy season which typically lasts from November to March. This study was conducted towards the end of the rainy season and during the onset of the 2021 dry season. Study clusters were created to contain approximately 175 households each, and included a minimum 100-m-wide buffer zone around each cluster core zone. ASB stations were installed on eligible structures found within the entire cluster area, including the buffer zones, while mosquito surveillance was performed only in the core zones.

### Study design

This study employed a crossover study design, utilizing the same clusters over two evaluation phases to efficiently assess feeding rates when either 2 or 3 ASB stations per eligible structure were deployed. After 2 weeks of pre-ASB deployment surveillance (01 February to 14 February 2021), study clusters were randomized 1:1 into two study arms: one arm that initially received 2 ASB stations per eligible structure and one arm that initially received 3 ASB stations per eligible structure (Fig. 2). The first phase of the study began with the initial ASB hang-up campaign during the weeks of 15 and 22 February 2021, with principal entomological surveillance beginning on 01 March 2021, approximately 7 days after ASB installation, and lasting for 6 weeks. At the midway point of the study (from April 12 to 16, 2021), crossover activities ensured that eligible structures from the first group of clusters received one additional ASB station per eligible structure (transitioning from 2 to 3 ASB stations) while one ASB station was removed from each eligible structure in the second group of clusters (transitioning from 3 to 2 ASB stations) (Fig. 1). Post-crossover mosquito surveillance resumed on 19 April 2021, and lasted an additional 6 weeks. The removal of ASB stations from the study areas were conducted between 31 May and 6 June 2021.

The spatial density and weighted spatial density per hectare of eligible structures and of installed ASB stations (Table 1) were calculated using procedures adapted from Ottensmann [22, 23]. Briefly, North–South and East–West gridlines were superimposed upon a map of each cluster, with the parallel lines separated by 100 m, dividing the area into 1-hectare squares. Density refers to the number of eligible structures (or ATSB stations installed) per hectare within the boundaries of each cluster, while the weighted density refers to the average density of eligible structures (or ASB stations installed) weighted by the number of eligible structures per grid square with at least on eligible structure.

**Table 1:** The spatial density and weighted spatial density of eligible housing structures and ASB stations per hectare in the study clusters

Cluster ID	Total eligible structures	Eligible structures per hectare	Weighted eligible structures per hectare	ASB stations in study phase 1				ASB stations in study phase 2			
				Target number per eligible structure	Total ASB stations	ASB stations per hectare	Weighted ASB stations per hectare	Target number per eligible structure	Total ASB stations	ASB stations per hectare	Weighted ASB stations per hectare
13	283	3.41	4.93	2	433	4.88	6.27	3	588	6.61	8.49
77	298	3.53	5.27	2	480	5.35	7.79	3	701	7.82	11.16
163	363	3.02	5.61	2	456	3.12	4.10	3	603	4.18	5.56
143	250	2.68	5.67	2	301	2.80	4.38	3	431	3.90	6.18
45	513	2.89	4.24	2	979	4.58	6.01	3	1208	5.62	7.38
33	280	3.15	4.83	3	592	6.07	8.55	2	401	3.96	5.61
151	322	2.95	4.49	3	665	5.28	7.69	2	476	3.73	5.29
84	247	3.28	4.83	3	469	5.37	7.33	2	311	3.55	4.71
194	205	3.12	4.97	3	611	7.43	11.12	2	181	1.98	3.07
158	202	2.58	3.72	3	567	6.72	9.53	2	419	4.90	7.33

### ASB stations

The product evaluated was the Sarabi v1.1.1 ASB station. The bait matrix in the stations did not include insecticide, which is the key difference between a standard ATSB and the prototype ASB used here. These prototype ASBs stations did include 0.8% uranine as a dye, which allows for the detection of mosquitoes that have acquired a sugar meal from the ASB using UV fluorescence light microscopy. Additional components of the bait matrix included date syrup (as a source of sugar and a mosquito attractant), potassium sorbate and sodium benzoate (as preservatives), and denatonium benzoate (Bitrex^®^), to deter human consumption of the bait). Pre-study evaluations in outdoor cages in Kenya had demonstrated that uranine fluorescence was detectable for a mean of 4.5 days in *Anopheles* mosquitoes which had fed on ASB stations with 0.8% uranine.

### Community sensitization and consent to participate

Sensitization meetings with community leaders were held prior to installation of ASB stations and the baseline collections of mosquitoes. Community leaders were identified through the chief, the local health facility staff, and community health volunteers affiliated with the facility. There was one meeting in each village, organized by the ATSB research and field study teams, the district health office and local health facility environmental health officers (EHTs), and community health workers. Due to COVID-19 mitigation measures which limited community meeting sizes, public address systems were also used to communicate key messages across the ASB clusters. Prior to household mosquito sampling and distribution of ASB stations, written voluntary informed consent was obtained from the household heads of every home to be enrolled into the study for permission to deploy ASB stations and collect household variables such as house construction, vector control use, and demographic data. Explanation and appropriate communication regarding buffer areas were emphasized as buffers created situations where some residents in the same village received bait stations and others did not. For the sampling of mosquitoes, oral consent was obtained prior to each collection.

### ASB installation

The installation of ASB stations on household structures in the study areas was not random but based on a standard criterion. Structures that were eligible to receive ASB stations were defined as those with (1) a complete roof; (2) walls at least 1 m high; (3) at least 3 complete walls. At each eligible structure, ASB stations were hung using bamboo sticks, nails, and wires. ASB stations were hung approximately 1.6–1.8 m above the ground (where possible) on an exterior house wall, prioritizing protected locations under an eave or roof overhang (Fig. 1).

### ASB monitoring

In each cluster, two study technicians were trained to monitor the ASB stations using a standardized questionnaire on Samsung tablets. Bait stations that were damaged, leaking, or observed to have a high degree of mold growth were removed and replaced with a fresh ASB station monthly to ensure high coverage. ASB stations that were missing, were also replaced, with the permission of residents, to further maintain coverage. During the monthly monitoring visits, photos of bait stations, present on them, were taken.

### Mosquito sampling

Eligible households that provided consent to participate in mosquito surveillance activities were entered into a master list which was used for random selection of households for each collection night. Within each cluster, mosquitoes were sampled from 12 houses over 15 nights every month for 720 total collection nights per cluster (360 during phase 1, prior to the crossover, and 360 during phase 2). During each collection, mosquitoes were sampled overnight from 18:00 h to 06:00 h, both indoors and outdoors at the same households using CDC Miniature Downdraft Blacklight UV Light Traps (CDC LTs) (Model 912, John W. Hock Co., Gainesville FL). For outdoor collections, CDC LTs were set at least 5 m, but no more than 10 m, from the entrance to an eligible structure with at least one sleeping space, at 1.5 m from the ground. For the indoor collections, the main sleeping space of each eligible structure was identified, and CDC LTs were placed 1.5 m high at the foot of an occupied bed net. Members of the households were instructed not to touch or move the traps.

### Mosquito collections

Primary sampling occurred from 01 March through 09 April, 2021 (Phase 1) and from 19 April to 28 May, 2021 (Phase 2). The sampling strategy was informed by mosquito densities observed during preliminary collections from 01 to 14 February, 2021, prior to ASB installation and supported the minimum assumption of 1.5 adult female vectors per collection night. The strategy was, therefore, designed to ensure the collection of a total of at least 300 adult females of each vector species from each cluster, both before and after crossover, to obtain sufficient samples to estimate a daily feeding rate of 7.5% with a precision of 5%, thus having feeding rates between 2.5% and 12.5% (Confidence interval for one proportion in a Cluster Randomized Design; PASS^®^ 14, NCSS, LLC, Kaysville, UT). Further details of the sample size calculation formula are provided in the sample size considerations section.

### Mosquito processing

All mosquito collections (trap cups and cages) were labelled with a unique collection barcode ID for which the collection date, household/structure barcode ID, and indoor or outdoor location were recorded using the CommCare Data Collection App (Dimagi Inc., Cambridge, USA).

Upon collection each morning, labeled trap contents were transported to the central field laboratory in Kaoma town and killed in a −20 °C freezer. All anopheline mosquitoes were screened for presence of uranine dye in the abdomen, as an indicator of feeding from an ASB, using a UV Leica Stereo Fluorescence microscope MZ10F model (Leica Microsystems Ltd, Switzerland). Thereafter, each specimen was morphologically identified using the standard key [24]. Each anopheline mosquito was then classified based on the abdominal appearance; unfed, partly fed, fed, or gravid according to World Health Organization (WHO) standards [25]. Each morphologically identified anopheline specimen was assigned a sample ID and placed individually in 1.7 millilitre Eppendorf tubes (Sigma co. Ltd) over silica gel and stored at −20 ℃ until further processing.

A representative subsample of just over 7,000 mosquitoes was further selected for PCR species identification, PCR blood meal analysis, and ELISA sporozoite screening. Samples were randomly selected from among pools designed to ensure a minimum of 2,500 *Anopheles funestus*, 1,300 *An. gambiae*, and 1,000 each of *Anopheles coustani, Anopheles squamosus*, and other *Anopheles* spp., balanced across study weeks, study clusters, and trap location. For species identification, DNA was extracted from the specimen’s abdomen, legs, and/or wings and amplified using the appropriate *An. gambiae* [26] and/or *An. funestus* [27] assays. For blood fed mosquitoes, DNA extracted from the bloodmeal within the mosquito abdomen was used to identify the blood meal hosts using PCR and gel electrophoresis [28]. The head and thorax of each specimen was then tested for the presence of *Plasmodium falciparum* circumsporozoite protein using standard sandwich ELISA methods [29, 30].

### Converting proportions of dye-positive mosquitoes to daily feeding rates

The proportion of mosquitoes collected that were positive for uranine dye was converted to an estimated daily feeding rate using an Imperial College, London mathematical model expressed as a polynomial equation of the form F = au^2^ + bu, where F is the proportion positive for uranine and u is the daily feeding rate [21]. The coefficients a and b varied according to the feeding rate range (up to 10% daily or 10–50% daily) and were based on the previously measured uranine dye longevity of 4.5 days with standard deviation of 0.5 days assumed. (To derive u, the formula was expressed as u = [sqrt{(b^2^) + (4aF)}—b]/[2a]). The model also calculated upper and lower estimates of daily feeding rates and assumed a natural daily mortality of 9.6%, no excess mortality caused by the uranine dye, a constant mosquito population, and an additional 9% mortality caused by standard vector control interventions (IRS and ITNs). When investigated mathematically, the proportion of mosquitoes positive for uranine as a function of feeding rate varied only slightly for a variable mosquito population, hence the assumption of a constant mosquito population for simplicity.

### Sample size considerations

To calculate the number of clusters required to accurately estimate a mosquito feeding rate of 7.5% per night, with a targeted 5% half width of confidence interval, an R function was written) to implement the formula for “Confidence interval for one proportion in a Cluster Randomized Design” as implemented in PASS software.

$$K={\left(\frac{{Z}_{\left\{1-\frac{\alpha }{2}\right\}}\left(\sqrt{P(1-P}\right)}{d}\right)}^{2}{\left(\frac{{Z}_{\left\{1-\frac{\alpha }{2}\right\}} \left(\sqrt{P(1-P}\right)}{d}\right)}^{2}\left\{\frac{(1-\rho )}{M} + \rho + \rho \times {C}^{2}\right\}$$

The formula calculates the required number of clusters (*K*) for a targeted proportion (of feeding) at a desired confidence interval width (precision) for a range of potential scenarios. The different terms of the formula are:

1. *K* is the number of clusters required
2. ${Z}_{\left\{1-\frac{\alpha }{2}\right\}}$ is the quantile of the normal distribution that controls for a standard 5% false positive rate
3. *P* is the population proportion of female *Anopheles* mosquitoes that are positive for uranine. This proportion is computed from the feeding rate using a second order polynomial
  $$P=-2.2688\times {f}^{2} + 2.4951\times f + 0.0484$$

based on previous laboratory studies measuring the duration of detection of Uranine in mosquitoes.

4. The term *d* is half the Width of the Confidence Interval (WCI) associated with the feeding rate, that is precision. The WCI was chosen such that the lower confidence limits were at or above the threshold feeding rate of 2.5%. Note that, the narrower the WCI, the more precise the estimate of feeding rate will be. Narrower WCI lead to greater cluster requirements.
5. The ICC (intra cluster correlation) is *rho* (ρ), set at 10%.
6. *M* is the cluster size. The cluster size is a function of mosquito availability (an average of 1.5 *Anopheles* adult female mosquitoes collected per household per night was assumed) and sampling effort.
7. *C* is the coefficient of variation of cluster size, computed as the ratio between the standard deviation of the cluster sizes to the mean of cluster size, set at 0.4.

The biological assumptions involved are as follows:

- Average no. female adult vectors caught/household/night = 1.5
- ICC is between clusters, not between households within clusters
- Threshold daily bait killing rate for effectiveness = 2.5%
- Daily bait feeding rate on ASB = daily bait killing rate for ATSB
- Background = 60% LLIN and 25% IRS

Further assumptions included sampling from 10 households/cluster/night, 10 nights/month over 2 months in each cluster (300 adult female mosquitoes per cluster), C = 0.4 and ICC = 0.1. Results indicated that a minimum of 7 clusters was required to estimate a daily feeding rate of 7.5% (± 5%), which could indicate a daily feeding rate of or above 2.5% (the lower limit) considered to be sufficient to lead to a 30% reduction in malaria incidence.

### Meteorological data

Meteorological data was collected using a Davis Vantage Vue Wireless weather station positioned centrally at the field laboratory in Kaoma Town (S 14.82°, E 24.81°). During the study period, the onset of the dry season resulted in much cooler and dryer conditions. Weekly averages for maximum daily temperature, minimum daily temperature, and daily rainfall varied from 30.5 °C, 18.6 °C, and 23.6 mm during the first 3 weeks of the study to 28.4 °C, 8.9 °C, and 0.00 mm during the final 3 weeks.

### Data analysis

Data were maintained, cleaned, transformed, and summarized by descriptive statistics (including species compositions and abundance) using Microsoft Excel v2111 (Microsoft Corporation, Redmond, WA) and Tableau Desktop v2020.4.11 (Tableau Software LLC, Seattle, WA). Sata/SE 14.2 (StatCorp LLC, College Station, TX) and R and RStudio (version 4.1.2 and version 2022.7.1, respectively) were used for formal statistical analyses, including a linear regression analysis with robust standard errors clustered at the study cluster level to compare average vector ASB feeding rates and overall vector abundance by (1) indoor and outdoor trap locations and (2) by study arm. Additionally, Spearman’s ranked test was used to evaluate potential correlations between cluster-average feeding rates and crude ASB station density (the number of ASB stations per hectare within a cluster), weighted ASB station density (the number of ASB stations per occupied hectare within a cluster), as well as between overall weekly feeding rates and weekly rainfall amounts under various time-lag scenarios. Unless otherwise described, primary outcomes are presented as point estimates with 95% confidence intervals in parenthesis. For the linear regression analyses, P-values are from an F-test that calculates the difference between average feeding rates and estimates the probability that the actual difference equals zero. For Spearman’s rank tests, P-values are from a t-test that estimates the probability that the actual correlation coefficient equals zero.

## Results

During the entire 14 weeks of CDC LT surveillance (the 2-week pre-ASB deployment period from 01 to 14 February 2021 and the 12-week trial from 01 March to 28 May 2021), a total of 144,550 *Anopheles* spp. mosquitoes were collected over 14,859 collection-nights (Additional file 1: Table S1). The most abundant species collected was *An. funestus s.l.* (31% of the total; 45,038 specimens), followed by *An. squamosus* (20%; 29,033), *Anopheles tchekedii* (19%; 27,461), *An. coustani s.l.* (19%; 27,149), *Anopheles maculipalpis* (2%; 3,523), and *An. gambiae s.l.* (2%; 3,255). Other species encountered, each comprising less than 1% of the total and fewer than 1,000 specimens, included *Anopheles brunnipes*, *Anopheles rufipes*, *Anopheles gibbinsi*, *Anopheles pretoriensis*, *Anopheles implexus,* and *Anopheles pharoensis*. 5% of the *Anopheles* specimens collected (6,761) were not identified to species because of poor physical condition.

A subsample of specimens collected were screened for species confirmation, blood-meal hosts, and presence of *P. falciparum* sporozoites in the head and thorax. PCR species identification confirmed a total of 2,150 *An. funestus s.l.* specimens: of these, 97.2% (2,090) were *An. funestus *sensu stricto (*s.s*.), 2.6% (56) were *Anopheles parensis*, and very few were identified as *Anopheles rivulorum* (3) and *An. rivulorum*-like. Of the 1,238 confirmed *An. gambiae s.l.* specimens, 96.8% (1,199) were *Anopheles arabiensis*, 1.8% (22) were *An. gambiae s.s.,* and 1.4% (17) were *Anopheles quadriannulatus*. *Anopheles coustani s.s.* was the only species identified from the Coustani group (586 specimens).

Results of the sporozoite screening, presented in Table 2, indicate that the dominant vector species in the study area is *An. funestus s.s*., with an overall sporozoite rate of 3.16% and accounting for 94% of all sporozoite-positive mosquitoes. *Anopheles arabiensis* is likely an important secondary vector, and *An. squamosus* may play a minor role in transmission as well. The most common blood-meal source identified was human (44%), while cow (32%) and goat (23%) were also common and the human blood index (percentage of all blood meals that were human) varied substantially by species (Table 2).

**Table 2:** Results of *Plasmodium falciparum* ELISA screening and human blood indices

	*P. falciparum* ELISA results				
Species^1^	Negative	Positive	Sporozoite Rate (%)	% of all sporozoite positives	Human Blood Index^2^
*An. funestus s.s*	2,024	66	3.16	94.3	0.70
*An. arabiensis*	1,042	2	0.19	2.9	0.65
*An. squamosus*	977	2	0.20	2.9	0.06
*An. coustani*	575	0	0.00	–	0.13
*An. gambiae s.s*	22	0	0.00	–	1.00
*An. leesoni*	56	0	0.00	–	0.31
*An. maculupalpis*	795	0	0.00	–	0.02
*An. parensis*	56	0	0.00	–	0.09
*An. pretoriensis*	39	0	0.00	–	0.00
*An. quadriannulatus*	13	0	0.00	–	0.00
*An. rivulorum*	3	0	0.00	–	0.50
*An. rivulorum-like*	1	0	0.00	–	0.00

^1^Of the 70 total sporozoite positive samples, 5 did not produce amplicons with mosquito species identification primers. In this table, these specimens were included on the basis of their corresponding morphological IDs: 4 *An. funestus* s.l. and 1 *An. gambiae* s.l

^2^Proportion of all blood meals that were human

Subsequent results and analyses presented here focus on *An. funestus s.l*. and *An. gambiae s.l.* as likely the most important vectors in the study setting.

### Primary vector abundance by cluster and trap location

During the 12 weeks of mosquito surveillance following ASB station installation, there were 12,124 distinct CDC LT collections recorded. There were 42,587 *An. funestus s.l.* and 1,940 *An. gambiae* s.l. specimens collected. As expected given the collection methods, the vast majority of specimens from each species group were female: 96.2% of *An. funestus* (40,949/42,587) and 98.2% of *An. gambiae* (1,906/1,940).

**Fig. 3:**
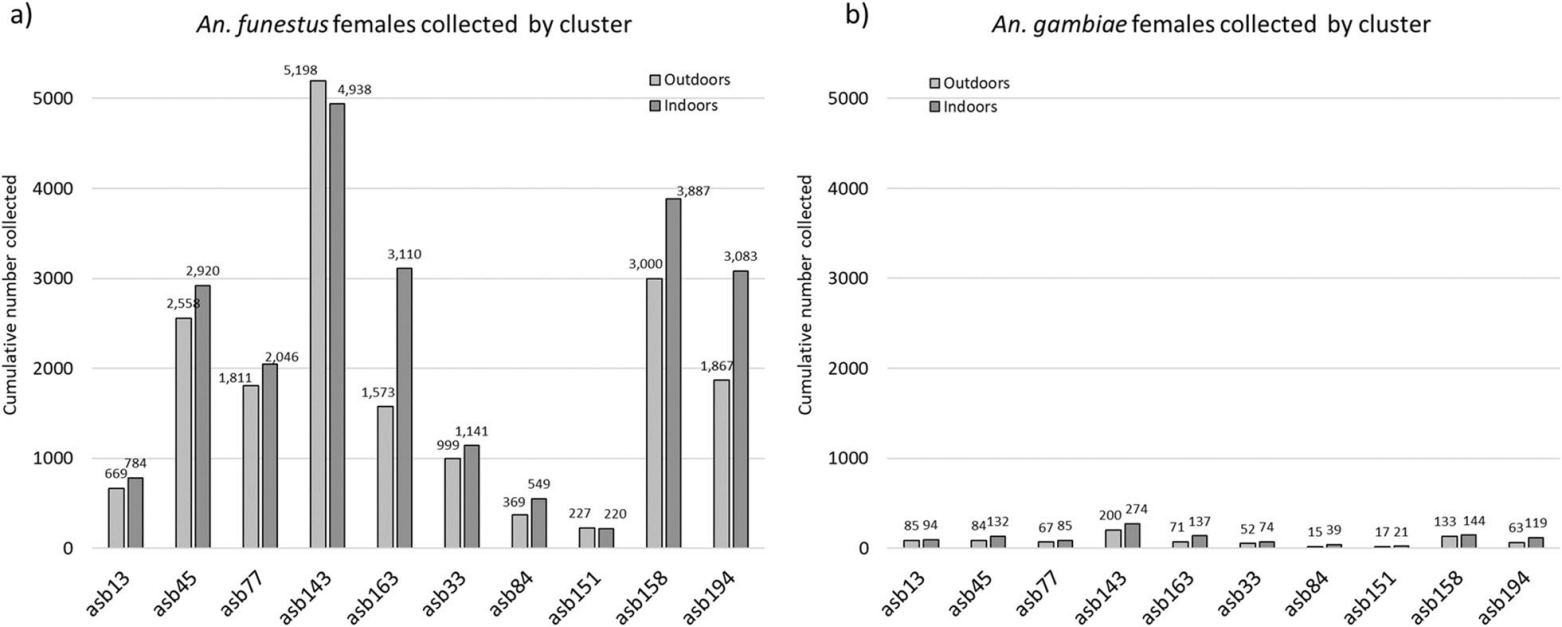
The overall abundance of **a** *An. funestus* and **b** *An. gambiae* female mosquitoes collected over the 12-week evaluation, by cluster

The overall abundance of each vector species by cluster is shown in Fig. 3. There was substantial variation in mosquito abundance observed by cluster, with a range for *An. funestus* from 5,198 to 227 (Coefficient of variation [CV] = 0.73) and for *An. gambiae* from 200 to 15 (CV = 0.65). However, the patterns in cluster-level abundance variation for each species were very tightly correlated (Spearman’s rho = 0.95, p < 0.0001).

The indoor to outdoor collection ratio for *An. funestus* was 1.24 (CI_95_ 0.97–1.51), indicating a slight, but not statistically significant, tendency to collect more mosquitoes indoors than outdoors. For *An. gambiae*, this tendency to collect more indoors compared to outdoors was significant, with an indoor to outdoor ratio of 1.42 (Cl_95_ 1.19–1.65).

### Proportions of primary vectors that were uranine positive

Overall, 21.6% (9,218/42,587) of *An. funestus* specimens collected and 10.4% (201/1,940) of *An. gambiae* specimens collected were positive for uranine in the abdomen. For both species, the proportion of uranine positive was higher in male mosquitoes, 31.2% (511/1,638) for *An. funestus* and 20.6% (7/34) for *An. gambiae*, than in female mosquitoes, 21.3% (8,707/40,949) for *An. funestus* and 10.2% (194/1,906) for *An. gambiae.* While the overall number of male mosquitoes collected was quite low, it is thought that high bait station feeding rates among males could potentially augment the effectiveness of an ATSB intervention. However, existing modeling [21] considers the impact on females vectors only. Therefore, further results and analysis presented here focus on the female mosquitoes only, unless otherwise noted.

**Fig. 4:**
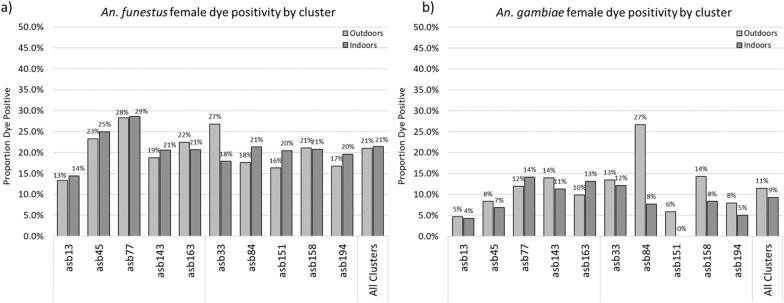
The proportions of female **a** *An. funestus* and **b** *An. gambiae* that were positive for uranine during the feeding validation study, by cluster

The overall proportion of female vectors that were positive for uranine (Fig. 4) also varied by cluster, though to a lesser degree than the variation observed in vector densities. The ranges of proportion dye-positive by cluster, considering indoor and outdoor collections together, for *An. funestus* was 13.9–28.5% (CV = 0.19) and was 2.6–13.2% for *An. gambiae* (CV = 0.42). Compared to the statistical assumption of an ICC of 10% used in the sample size calculations described above, the observed ICC for the proportion of mosquitoes feeding on ASBs was 8.6% for *An. funestus* and 23.3% for *An. gambiae.*

**Fig. 5:**
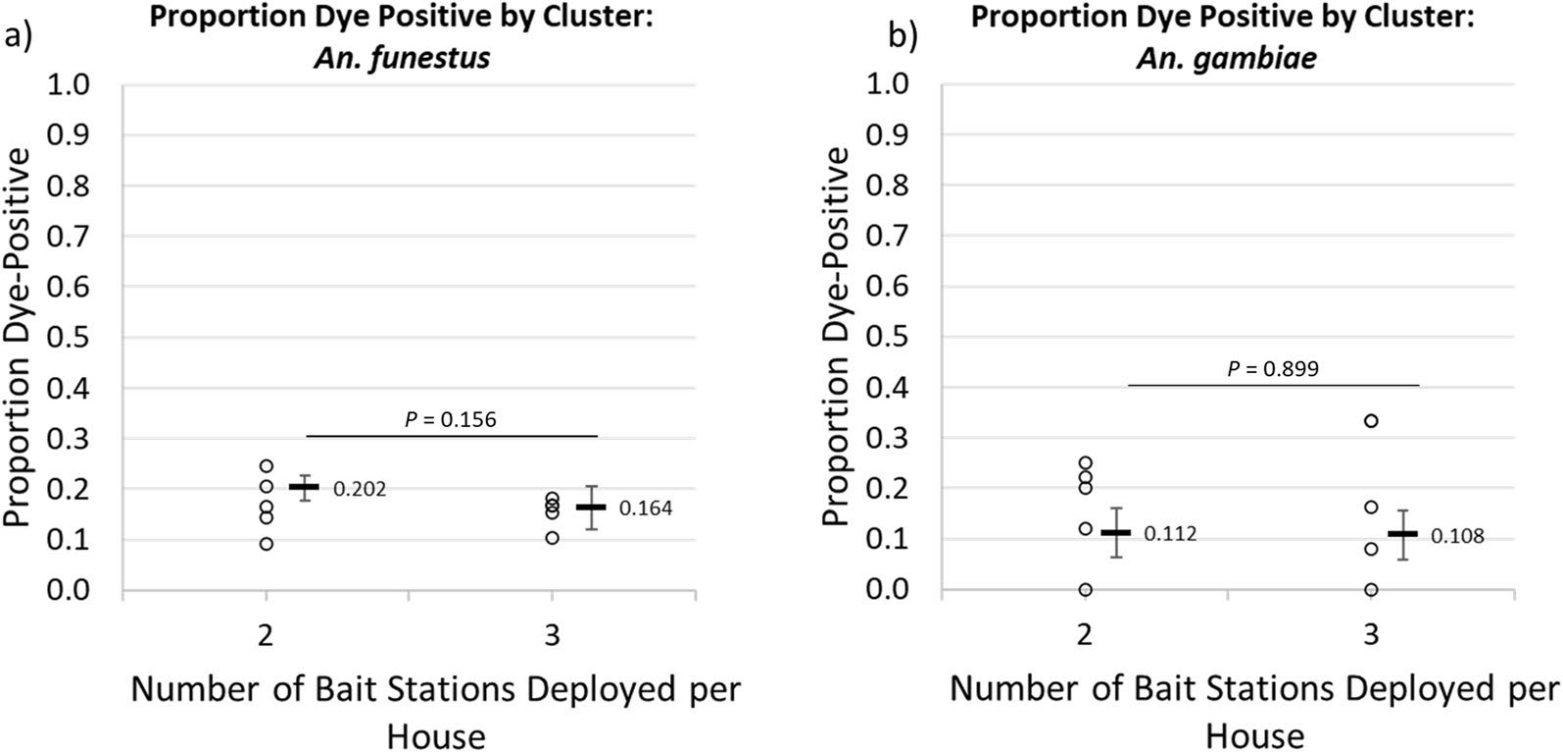
Differences in the proportions of female vectors that were positive for uranine among those collected indoors and outdoors for **a** *An. funestus* and **b** *An. gambiae*

To determine whether the proportion of female vectors positive for uranine was different among clusters where 2 ASB stations were hung per eligible structure compared to clusters where 3 ASB stations were hung per eligible structure, clusters were stratified by the number of ASB stations installed per eligible structure—accounting for crossover—and average dye positivity rates were compared using a clustered linear regression approach. No difference in proportions fed was observed between clusters with 2 or 3 ASB stations per eligible structure (Fig. 5), for either *An. funestus* (20.2% vs. 16.4%, a difference of 3.8% [−9.5 to 17%, *p* = 0.156]) or for *An. gambiae* (11.2% vs. 10.8%, a difference of 0.4% [−7.3 to 8.2%, *p* = 0.899]).

**Fig. 6:**
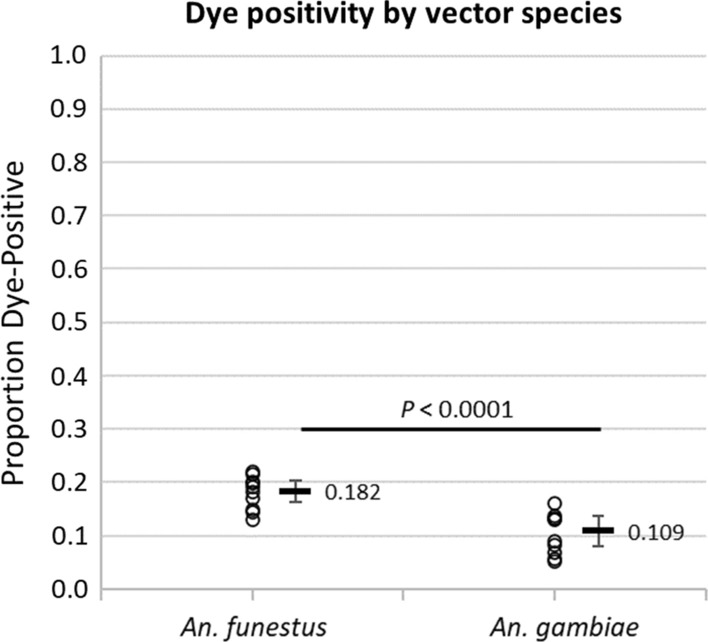
Differences in the proportions of female vectors that were positive for uranine among *An. funestus* and *An. gambiae*

**Fig. 7:**
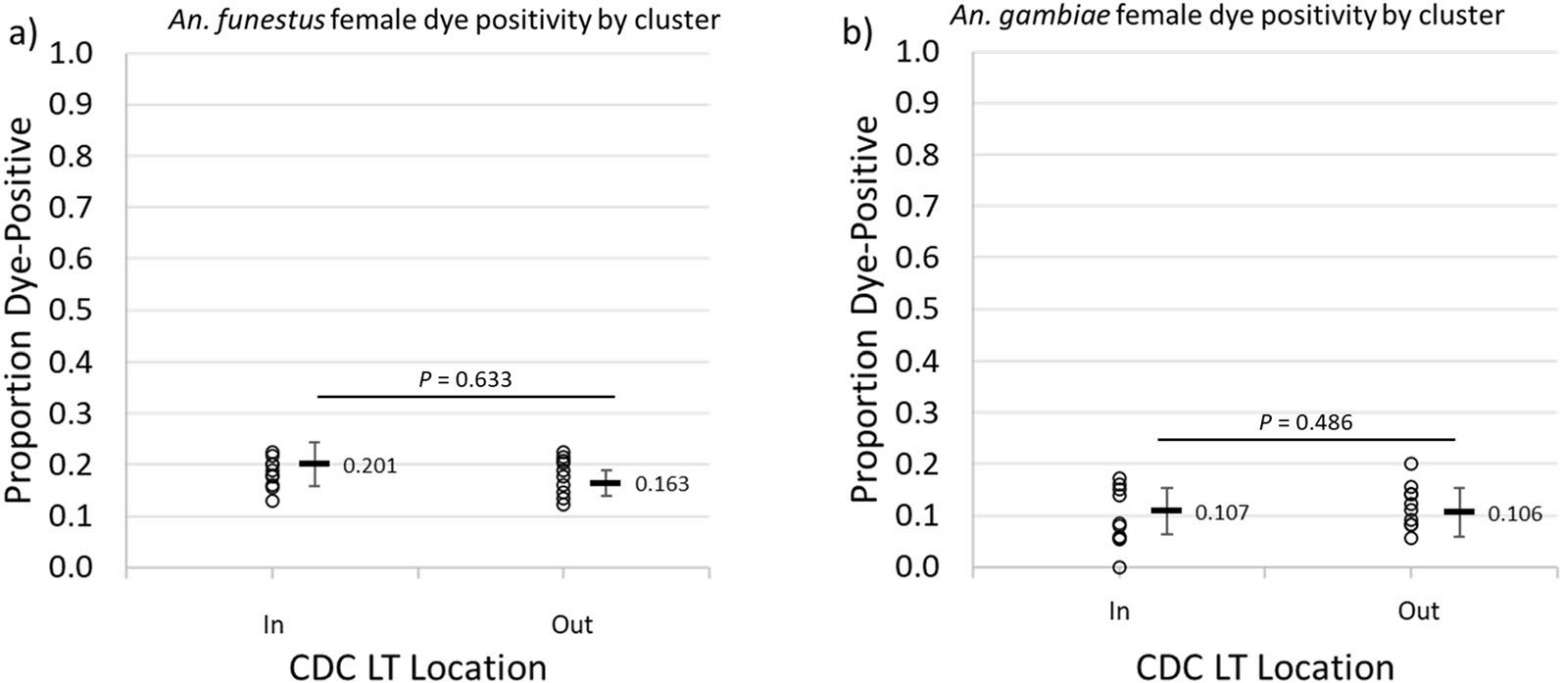
Differences in the proportions of female vectors that were positive for uranine among those collected indoors and outdoors for **a** *An. funestus* and **b** *An. gambiae*

Similar approaches were taken to determine if the proportions positive for uranine varied among vector species or among populations collected indoors and outdoors (Figs. 6 and 7). Among different species, a higher proportion of *An. funestus* females were positive (18.2% [1.2–20.2%]) compared to *An. gambiae* females (10.9, [8.8–13.36%]), a significant difference of 7.4% (5.2–9.5%, *p* < 0.0001) (Fig. 6). Among populations collected indoors versus outdoors, no significant differences were observed with either vector species (Fig. 7), and subsequent results and analyses presented here use data pooled from both indoor and outdoor trap locations.

### Estimates of daily feeding rates

**Fig. 8:**
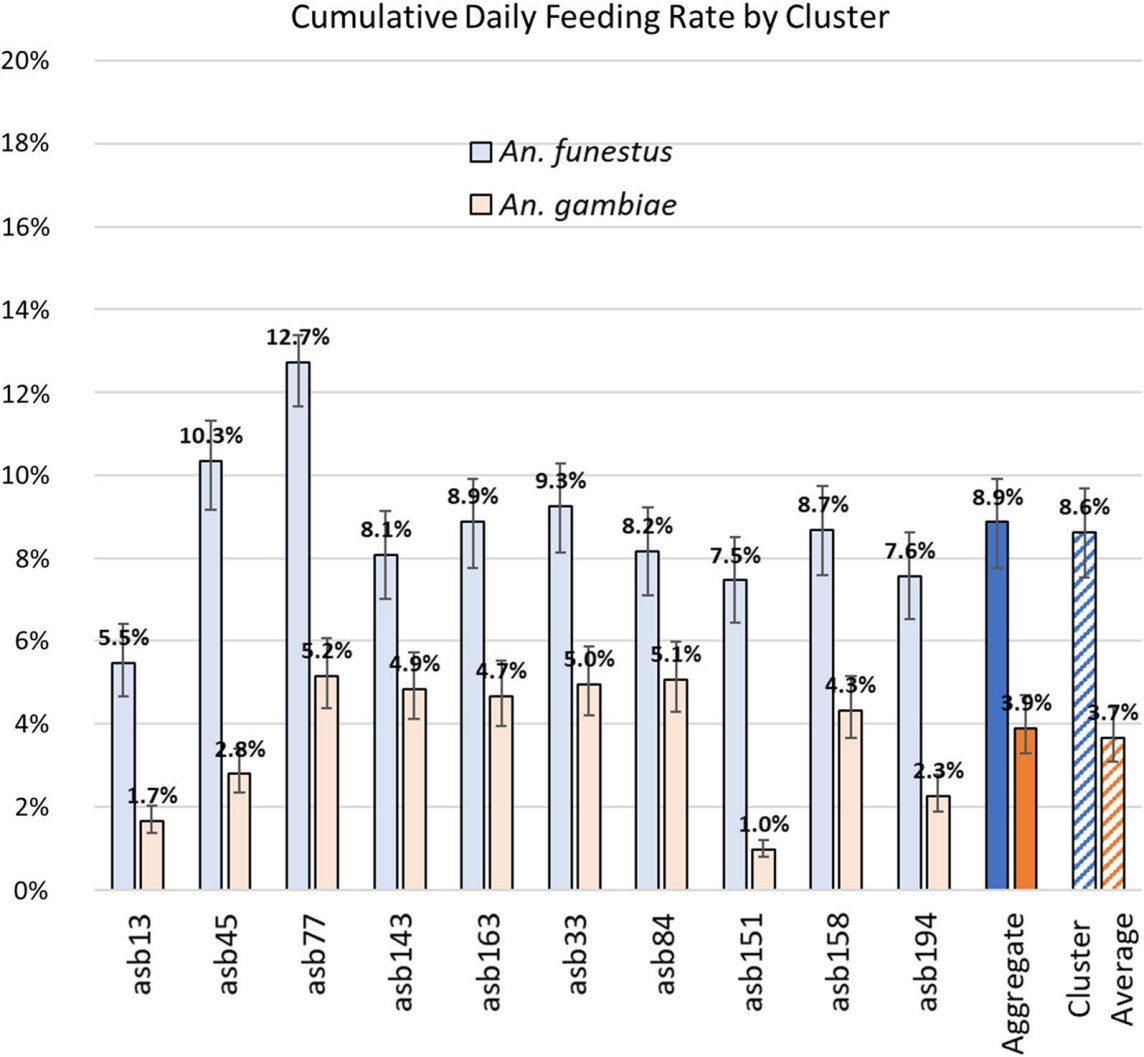
The estimated daily ASB feeding rates over the duration of the 12-week trial, calculated using the central assumptions of the primary model and presented by cluster

Using an Imperial College, London mathematical model expressed as a polynomial equation of the form described in the previous section, the proportion of each vector species sample that was positive for uranine dye was converted into estimated daily feeding rates (Fig. 8). Following the central assumptions of the main model, the overall aggregate feeding rate (total feeding rate for the duration of the 12-week study) for *An. funestus* was 8.9% (7.7–9.9%), with an inter-cluster range of 5.5% (4.7–6.4%) to 12.7% (11.7–13.4%) and a cluster average of 8.6% (7.5–9.7%). Estimated daily feeding rates for *An. gambiae* were lower, with an overall aggregate feeding rate of 3.9% (3.3–4.7%), an inter-cluster range of 1.0% (0.8–1.2%) to 5.2% (4.4–6.1%), and a cluster average of 3.7% (3.1–4.4%).

An alternate case scenario that assumed a lower rate of 5% for mortality caused by the other deployed vector control interventions was also utilized to estimate daily feeding rates in a somewhat less optimistic scenario. Results were similar, though with slightly reduced daily feeding rates: 8.0% (6.9–9.0%) overall aggregate daily feeding for *An. funestus* and 3.5% (3.0–4.2%) for *An. gambiae* (Additional file 1: Fig. S1).

### Estimates of daily feeding rates

Using an Imperial College, London mathematical model expressed as a polynomial equation of the form described in the previous section, the proportion of each vector species sample that was positive for uranine dye was converted into estimated daily feeding rates (Fig. 8). Following the central assumptions of the main model, the overall aggregate feeding rate (total feeding rate for the duration of the 12-week study) for *An. funestus* was 8.9% (7.7–9.9%), with an inter-cluster range of 5.5% (4.7–6.4%) to 12.7% (11.7–13.4%) and a cluster average of 8.6% (7.5–9.7%). Estimated daily feeding rates for *An. gambiae* were lower, with an overall aggregate feeding rate of 3.9% (3.3–4.7%), an inter-cluster range of 1.0% (0.8–1.2%) to 5.2% (4.4–6.1%), and a cluster average of 3.7% (3.1–4.4%).

An alternate case scenario that assumed a lower rate of 5% for mortality caused by the other deployed vector control interventions was also utilized to estimate daily feeding rates in a somewhat less optimistic scenario. Results were similar, though with slightly reduced daily feeding rates: 8.0% (6.9%–9.0%) overall aggregate daily feeding for *An. funestus* and 3.5% (3.0–4.2%) for *An. gambiae* (Additional file 1: Fig. S1).

### ASB feeding rates correlated with ASB density, and rainfall

Additional secondary objectives of this validation study included exploring whether ASB feeding rates varied in correlation to the density of ASB stations within a cluster (the number of ASB stations per hectare) or with rainfall (mm/week).

There were no statistically significant correlations observed between the average number of ASB stations per hectare and the daily feeding rates of either vector species (Additional file 1: Fig. S2). This was consistent whether using the average for each cluster of ASB stations per hectare that had ASB stations installed (which ranged from 1.98 to 7.82 ASB stations per hectare) or the weighted average (which ranged from 3.07 to 11.16 ASB stations per hectare) which takes into account the aggregation of structures. Potential correlations between weekly rainfall and vector abundance and ASB feeding rates were tested using four scenarios: no time lag between weekly rainfall amount and vector surveillance results, a 1-week lag, a 2-week lag, and a 3-week lag (Additional file 1: Fig. S3). For *An. funestus,* there were no correlations observed between weekly rainfall and either mosquito abundance or ASB feeding rates in any of the scenarios. For *An. gambiae*, both outcomes were somewhat positively correlated with rainfall, with the strongest relationship observed with no time-lag [Spearman’s rho correlation between rainfall and abundance = 0.697 (*p* = 0.0117), between rainfall and feeding rates = 0.529 (*p* = 0.0790)].

### Post-crossover addition of a fresh ASB stations on eligible structures

After the crossover addition of new ASB stations in half of the study clusters, a post-hoc secondary objective of assessing whether fresh bait stations resulted in increased feeding was considered. To examine this, a second approach using cluster aggregated proportions of mosquitoes that were dye-positive stratified by the number of ASB stations installed per eligible structure was calculated—but only with data collected during the second phase of the study, after the crossover. This was essentially a comparison of feeding rates across clusters with 2 older ASB stations per structure vs. clusters with 2 older ASB stations and 1 brand new ASB station per structure. Even with the new bait stations hanging, there was no difference in feeding rates across study arms observed for either vector species (Additional file 1: Fig. S4).

### ASB monitoring

Of the 6,824 ASB stations visited at least once during the duration of the study (Phase 1 & 2), 716 (10.5%) reported having damage, with 503 of the 716 damaged stations also reported as having leaks. Of the 6,824 ASB stations visited at least once during the duration of the study (Phase 1 & 2), 542 reported having mold growth. An independent review of photographs taken by ASB field monitors during Phase 1 was conducted in June 2021 to validate reporting of damage and mold. During Phase 1, field monitors reported damage of 123 ASB stations, whereas the independent reviewer (looking at the photographs) reported damage of 34 ASB stations. During Phase 1, ASB field monitors reported leakage in 99 ASB stations, whereas the independent observer reported leakage in 55 ASB stations. During Phase 1, ASB field monitors reported mold on 77 ASB stations, whereas the independent observer reported mold on 306 ASB stations. Overall, the ASB station monitoring and replacement protocols maintained a high level of compliance among participating households, as more than 82% of all ASB stations deployed were found in use and collected at the end of the study. The majority of those ASB station not collected were reported to have been removed and discarded by the households.

## Discussion

This ASB feeding validation study is part of a package of preliminary studies designed to validate the ATSB approach to vector control prior to the start of a larger, 2-year cluster randomized control trial with an active malaria case incidence cohort. The results presented here show that the dominant vector species in the study area, *An. funestus* s*.l.,* readily fed from the prototype Sarabi v1.1.1 ASB station with an overall daily feeding rate of around 8.9% (CI_95%_ 7.7–9.9%). *Anopheles arabiensis*, a likely secondary vector in the study area, also fed from the ASB stations, albeit at a lower daily feeding rate of around 3.9%. (CI_95%_ 3.3–4.7%) These estimated daily feeding rates exceed the target of 2.5%, which is associated by modeling with a potential minimum 30% reduction in malaria incidence, by a statistically significant margin.

Results also show that while ASB feeding rates varied substantially from cluster to cluster, they were consistent among mosquitoes sampled indoors and outdoors. Furthermore, similar feeding rates were observed with deployment of two versus three ASB stations per eligible structure, and rates were similar across the range of ASB spatial densities (from 3.07 to 11.16 ASB stations per hectare) observed across the 10 study clusters.

The high proportion of females in the samples caught in and around households, and the relative consistency in sugar feeding rates and high human blood meal indices observed within each cluster throughout this 12-week validation study, suggest that *An. funestus* and *An. arabiensis* were initially attracted to occupied houses within the study clusters in search of a blood meal, and that if unsuccessful in acquiring a blood meal they were successfully diverted to the ASB stations as an alternative. It could also be possible that host-seeking mosquitoes took a sugar meal prior to acquiring a blood meal, perhaps to replenish energy stores before completing the blood feeding process. Interestingly, mosquitoes that successfully fed on an ASB station were equally likely to have been sampled indoors or outdoors, indicating that these vector populations are predominantly opportunistic in the relative location of their blood-seeking, sugar-feeding, and resting locations as defined here—either indoors or immediately outdoors in the peri-domestic area. This could suggest potential for a well-balanced effect for similarly deployed ATSBs on the dominant vector species in western Zambia, complimenting the core vector control interventions to provide a substantial incremental effect.

While the ASB feeding rates observed here are somewhat lower than the feeding rates reported recently for *An. gambiae s.l.* (likely predominantly *Anopheles coluzzii*) during similar studies in Mali [14, 20], it is nonetheless encouraging to find the approach validated in a different, less arid region of sub-Saharan Africa with a second key malaria vector species. Indeed, the rates of feeding observed here, particularly for *An. funestus*, are within ranges that are expected to produce significant excess mosquito mortality, above that induced by standard vector control measures alone, to a degree that is likely to also produce significant reductions in malaria transmission [21].

This study had several limitations. A single UV fluorescence microscope for dye identification in mosquitoes was not sufficient to quickly handle the volume of mosquito samples that were collected and hence lengthening the time required for dye screening. This is in combination with some uncertainty around dye persistence in mosquito abdomens and whether this is consistent in laboratory and field conditions or across vector species, all of which could have implications on the modelled conversions between dye positivity rates and daily feeding rates. Another operational challenge encountered was the need to continually update the ASB monitoring forms and procedures throughout the duration of the study: overall, ASB field monitors were likely over-reporting damage to bait stations and under-reporting mold growth. Lastly, the COVID-19 mitigation steps required throughout, complicated the logistics for mosquito collections, bait station monitoring, and the breadth and depth of community engagement and sensitization meetings in the study areas. Strengths of the study included utilizing robust power calculations and a substantial sampling effort, including equal sampling indoors and outdoors in the event that sugar feeding varied by mosquito populations. Despite the additional COVID-19 complications, community leadership was continually engaged and interested in the study itself and its implications for further research and development of the ATSB paradigm, and community buy-in was generally good.

## Conclusion

This ASB feeding validation study demonstrated that *An. funestus* and *An. gambiae* vector populations in Western Province, Zambia each readily fed from the prototype Sarabi v1.1.1 ASB sugar bait station. Furthermore, the observed feeding rates are in line with those required for ATSB stations to achieve sufficient reductions in the proportion of older mosquitoes to lead to significant reductions in malaria transmission, when used in combination with conventional control methods (IRS or LLIN), compared to the use of conventional methods alone. As such, these results were used to support the decision to fully implement the ATSB cluster randomized controlled trial with epidemiological primary outcomes in Zambia, deploying 2 ATSB stations per eligible structure within each intervention cluster.

## Supplementary Information

**Additional file 1: Table S1.** Total number of mosquitoes collected during the trial. Morphological IDs are presented. **Figure S1.** Comparisons of the daily feeding rates calculated from the proportions of mosquitoes collected that were dye positive, based on (a) the base-case scenario for *An. funestus*; (b) the alternate-case scenario for *An. funestus*; (c) the base-case scenario for *An. gambiae*; and (d) the alternate-case scenario for *An. gambiae*. **Figure S2.** No association between ASB feeding rates ASB spatial density, either crude numbers of ASB stations per hectare or weighted average ASB stations per occupied hectare, for (a) *An. funestus *or (b) weighted numbers of ASBs per hectare. **Figure S3.** Correlations between weekly rainfall amounts and (a) *An. funestus *feeding rates (b) *An. funestus *abundance (c) *An. gambiae *feeding rates, and (d) *An. gambiae *abundance. **Figure S4.** Cumulative dye positivity by study arm, post-crossover.

## Data Availability

The annotated datasets and STATA and R code used in the analysis are available from the authors upon reasonable request.

## Abbreviations

ASB: Attractive sugar bait (without insecticide)
ATSB: Attractive targeted sugar bait (with insecticide)
WHO: World Health Organization
ITN: Insecticide treated net
LLIN: Long-lasting insecticidal net
IRS: Indoor residual spraying
GTS: Global technical strategy for malaria
PCR: Polymerase chain reaction
ELISA: Enzyme-linked immunosorbent assay
MoH: Ministry of health
NMEP: National malaria elimination programme
NTO: Non-targeted organism
