# Supplementary material for: Feeding rates of malaria vectors from a prototype attractive sugar bait station in Western Province, Zambia: results of an entomological validation study
Source: Malar J. 2023 Mar 1;22:70. doi: 10.1186/s12936-023-04491-9 (PMC9974387; doi:10.1186/s12936-023-04491-9)
Supplement: Supplementary file 1 — Additional file 1: Table S1. Total number of mosquitoes collected during the trial. Morphological IDs are presented. Figure S1. Comparisons of the daily feeding rates calculated from the proportions of mosquitoes collected that were dye positive, based on (a) the base-case scenario for An. funestus; (b) the alternate-case scenario for An. funestus; (c) the base-case scenario for An. gambiae; and (d) the alternate-case scenario for An. gambiae. Figure S2. No association between ASB feeding rates ASB spatial density, either crude numbers of ASB stations per hectare or weighted average ASB stations per occupied hectare, for (a) An. funestus or (b) weighted numbers of ASBs per hectare. Figure S3. Correlations between weekly rainfall amounts and (a) An. funestus feeding rates (b) An. funestus abundance (c) An. gambiae feeding rates, and (d) An. gambiae abundance. Figure S4. Cumulative dye positivity by study arm, post-crossover. [file 12936_2023_4491_MOESM1_ESM.pptx]

## Slide 1
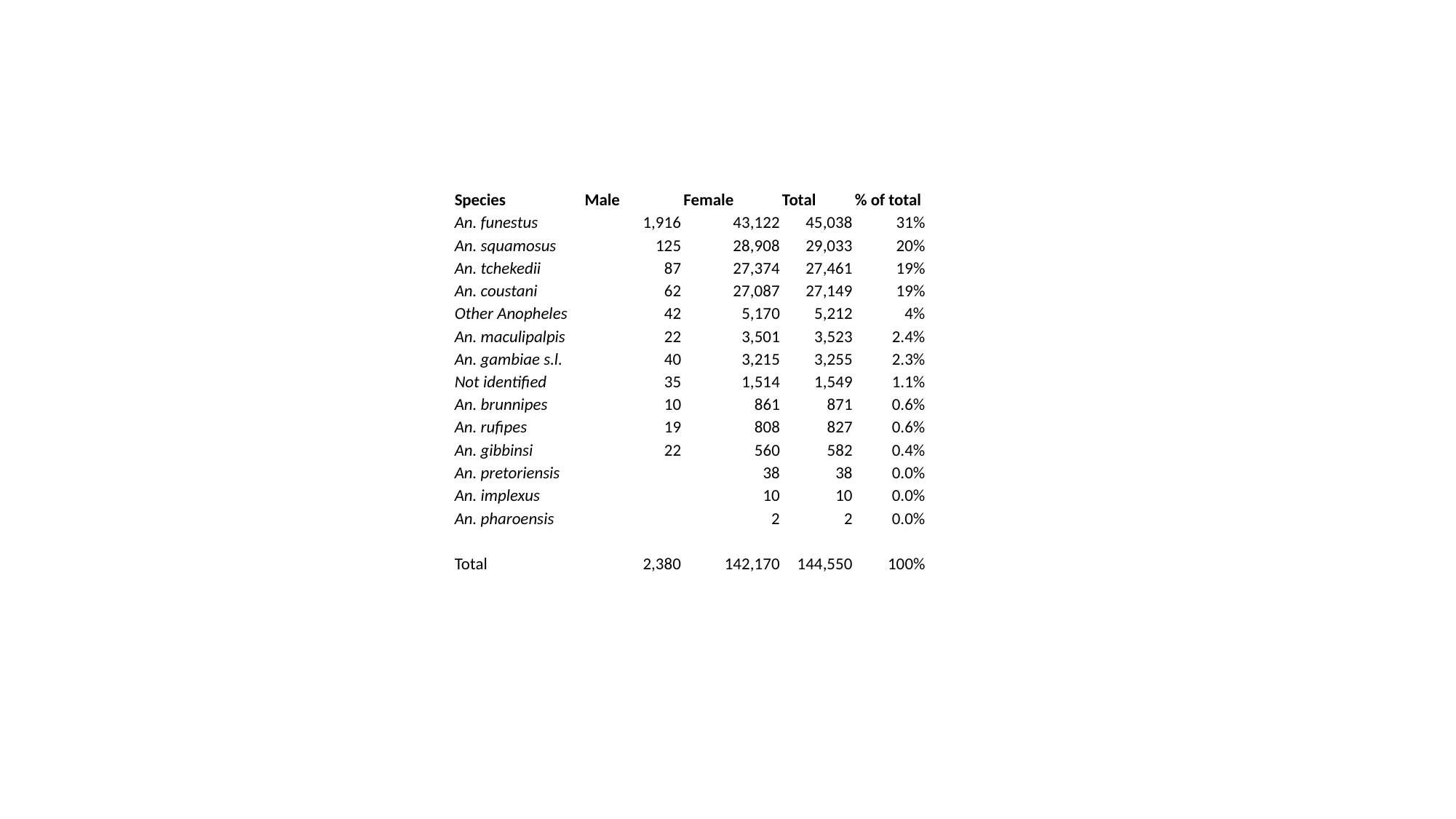

| Species | Male | Female | Total | % of total |
| --- | --- | --- | --- | --- |
| An. funestus | 1,916 | 43,122 | 45,038 | 31% |
| An. squamosus | 125 | 28,908 | 29,033 | 20% |
| An. tchekedii | 87 | 27,374 | 27,461 | 19% |
| An. coustani | 62 | 27,087 | 27,149 | 19% |
| Other Anopheles | 42 | 5,170 | 5,212 | 4% |
| An. maculipalpis | 22 | 3,501 | 3,523 | 2.4% |
| An. gambiae s.l. | 40 | 3,215 | 3,255 | 2.3% |
| Not identified | 35 | 1,514 | 1,549 | 1.1% |
| An. brunnipes | 10 | 861 | 871 | 0.6% |
| An. rufipes | 19 | 808 | 827 | 0.6% |
| An. gibbinsi | 22 | 560 | 582 | 0.4% |
| An. pretoriensis | | 38 | 38 | 0.0% |
| An. implexus | | 10 | 10 | 0.0% |
| An. pharoensis | | 2 | 2 | 0.0% |
| | | | | |
| Total | 2,380 | 142,170 | 144,550 | 100% |

## Slide 2
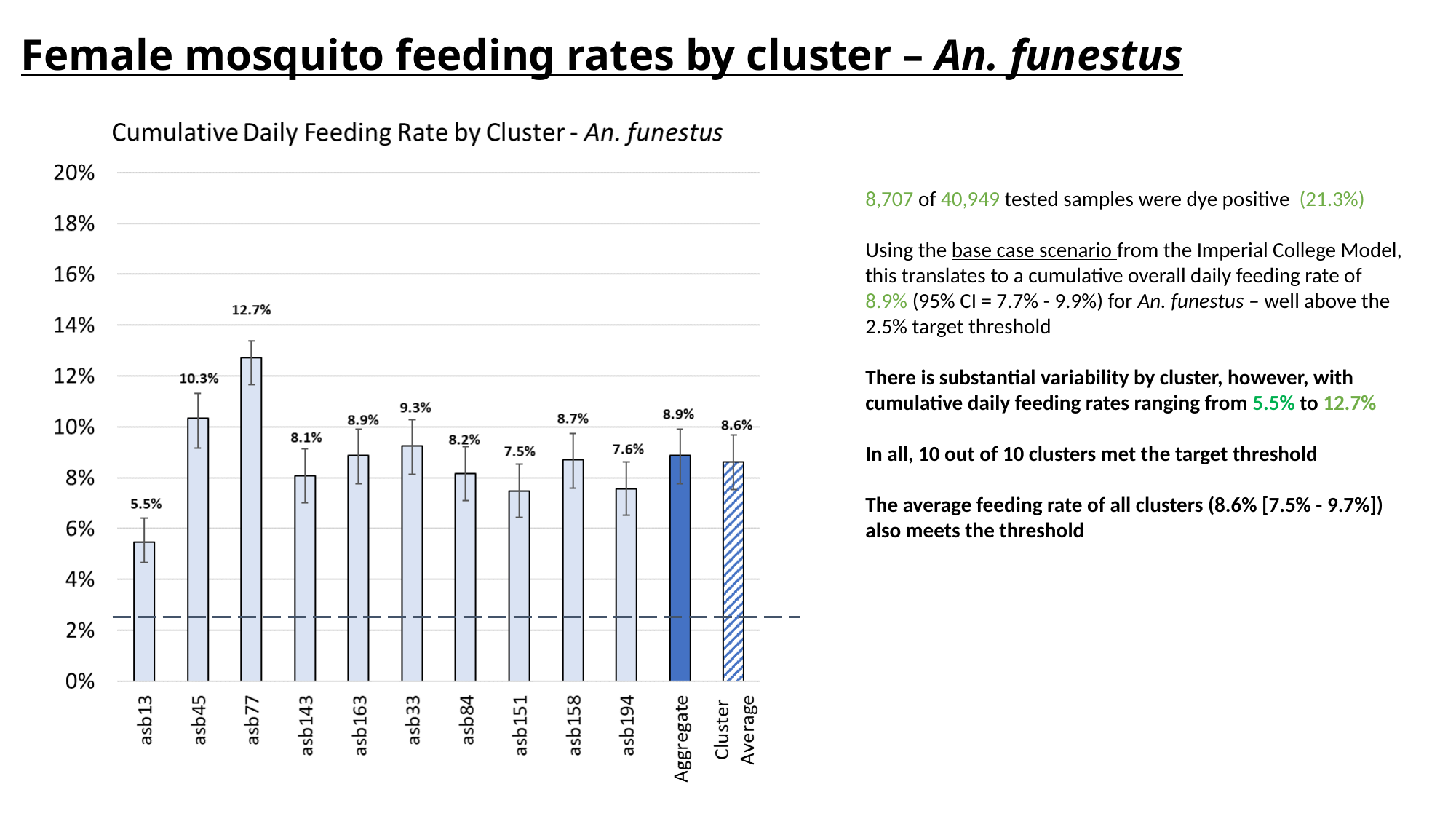

Female mosquito feeding rates by cluster – An. funestus
8,707 of 40,949 tested samples were dye positive (21.3%)
Using the base case scenario from the Imperial College Model, this translates to a cumulative overall daily feeding rate of 8.9% (95% CI = 7.7% - 9.9%) for An. funestus – well above the 2.5% target threshold
There is substantial variability by cluster, however, with cumulative daily feeding rates ranging from 5.5% to 12.7%
In all, 10 out of 10 clusters met the target threshold
The average feeding rate of all clusters (8.6% [7.5% - 9.7%]) also meets the threshold

## Slide 3
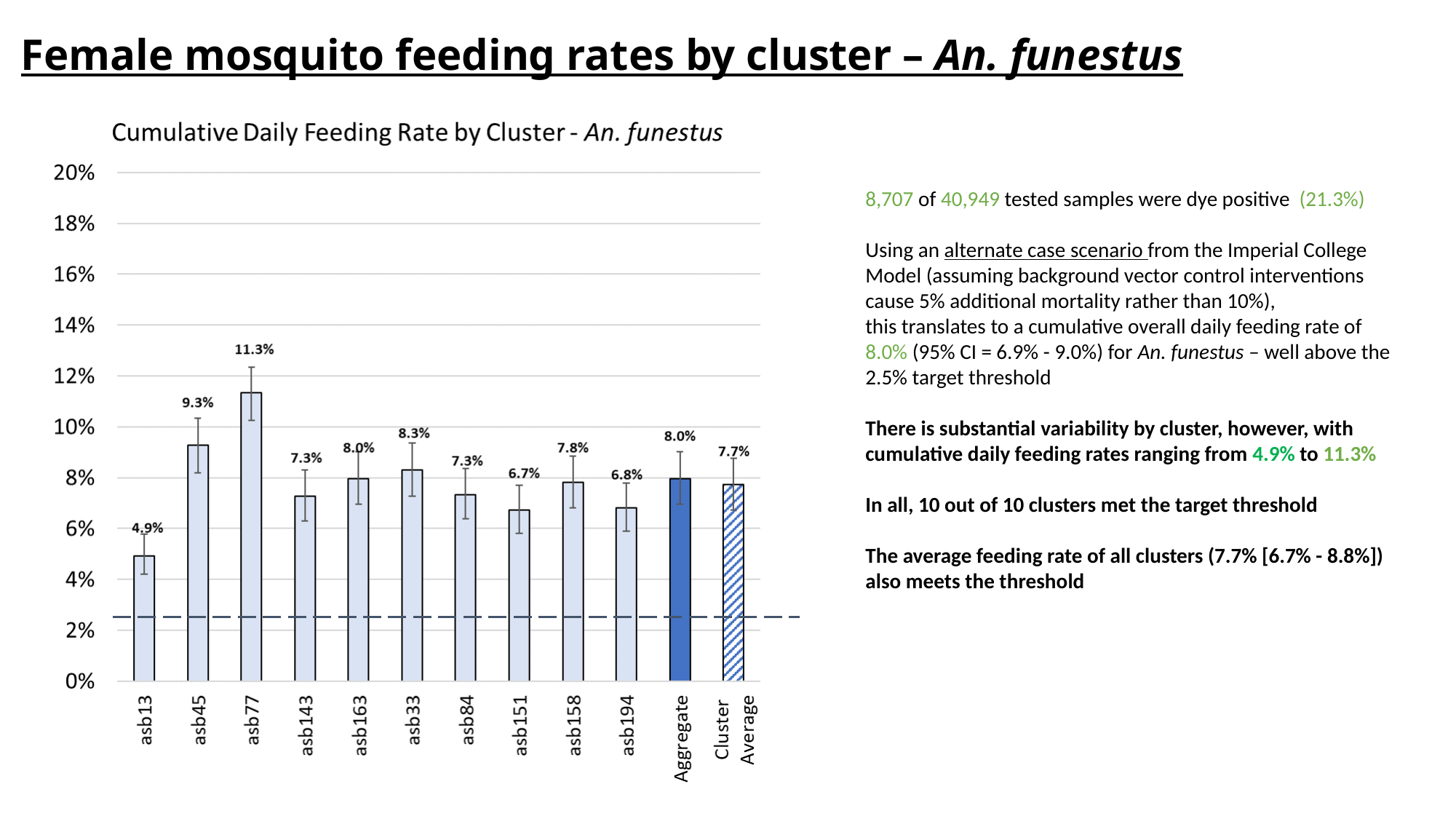

Female mosquito feeding rates by cluster – An. funestus
8,707 of 40,949 tested samples were dye positive (21.3%)
Using an alternate case scenario from the Imperial College Model (assuming background vector control interventions cause 5% additional mortality rather than 10%),
this translates to a cumulative overall daily feeding rate of 8.0% (95% CI = 6.9% - 9.0%) for An. funestus – well above the 2.5% target threshold
There is substantial variability by cluster, however, with cumulative daily feeding rates ranging from 4.9% to 11.3%
In all, 10 out of 10 clusters met the target threshold
The average feeding rate of all clusters (7.7% [6.7% - 8.8%]) also meets the threshold

## Slide 4
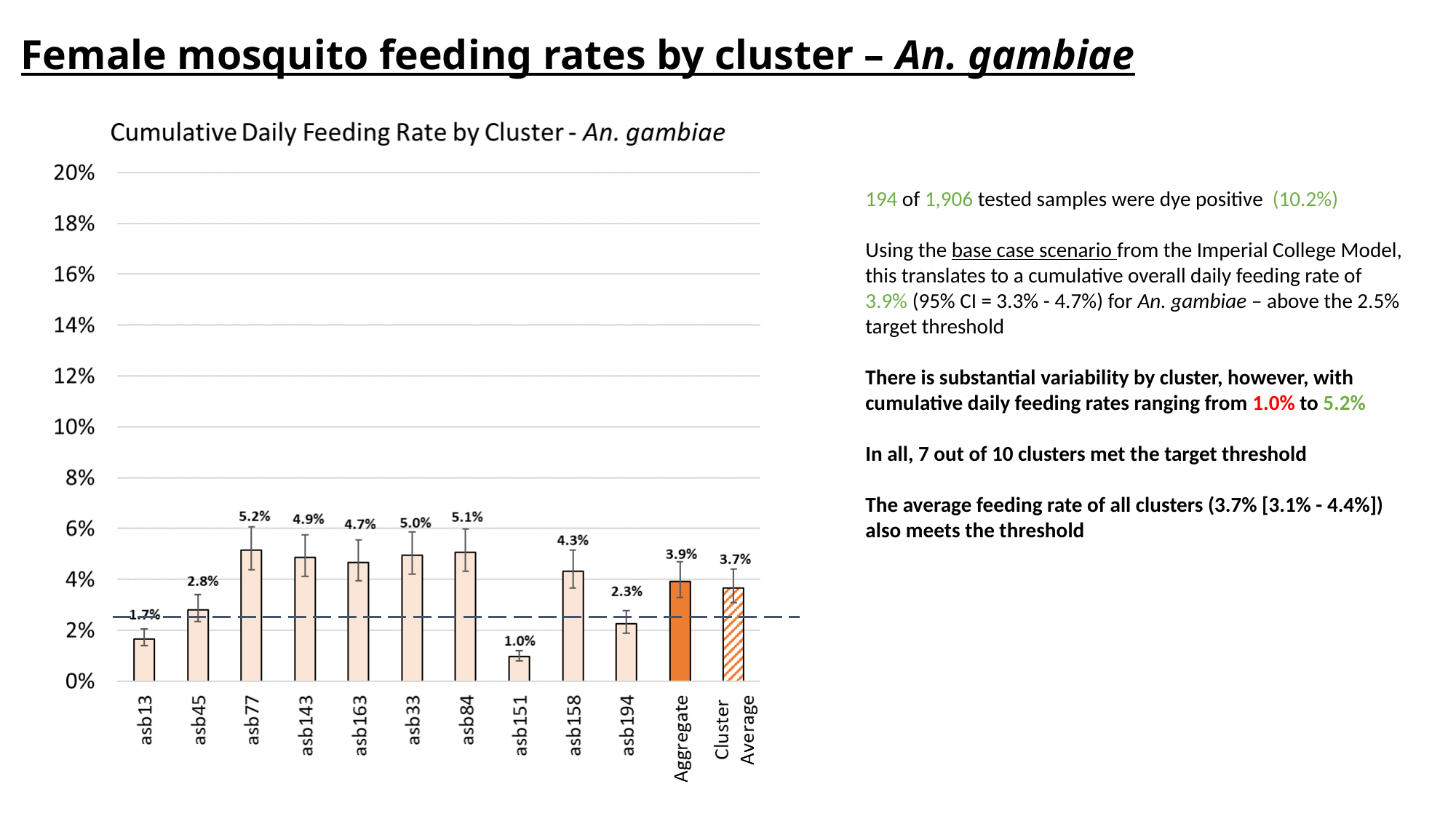

Female mosquito feeding rates by cluster – An. gambiae
194 of 1,906 tested samples were dye positive (10.2%)
Using the base case scenario from the Imperial College Model, this translates to a cumulative overall daily feeding rate of 3.9% (95% CI = 3.3% - 4.7%) for An. gambiae – above the 2.5% target threshold
There is substantial variability by cluster, however, with cumulative daily feeding rates ranging from 1.0% to 5.2%
In all, 7 out of 10 clusters met the target threshold
The average feeding rate of all clusters (3.7% [3.1% - 4.4%]) also meets the threshold

## Slide 5
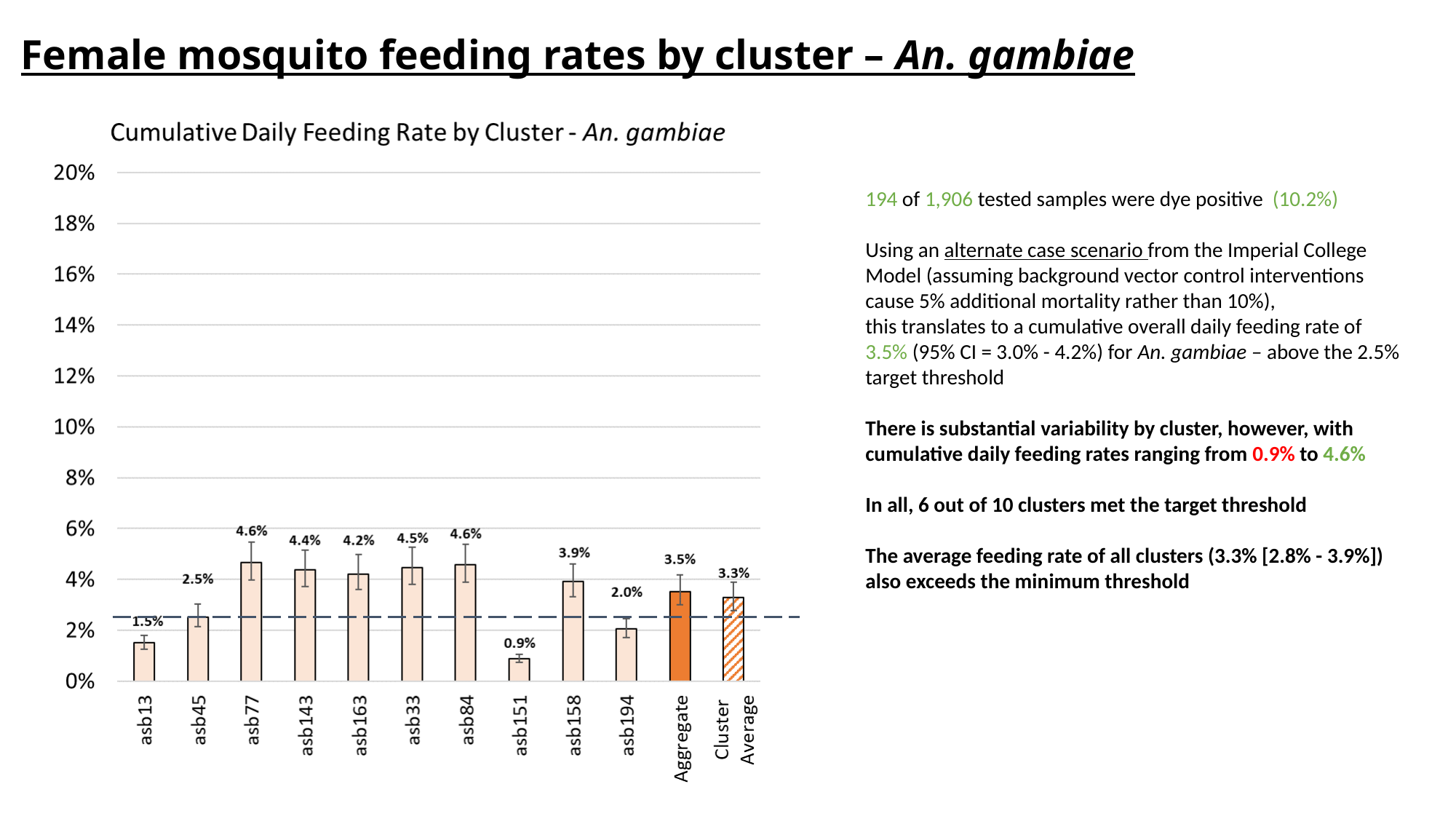

Female mosquito feeding rates by cluster – An. gambiae
194 of 1,906 tested samples were dye positive (10.2%)
Using an alternate case scenario from the Imperial College Model (assuming background vector control interventions cause 5% additional mortality rather than 10%),
this translates to a cumulative overall daily feeding rate of 3.5% (95% CI = 3.0% - 4.2%) for An. gambiae – above the 2.5% target threshold
There is substantial variability by cluster, however, with cumulative daily feeding rates ranging from 0.9% to 4.6%
In all, 6 out of 10 clusters met the target threshold
The average feeding rate of all clusters (3.3% [2.8% - 3.9%]) also exceeds the minimum threshold

## Slide 6
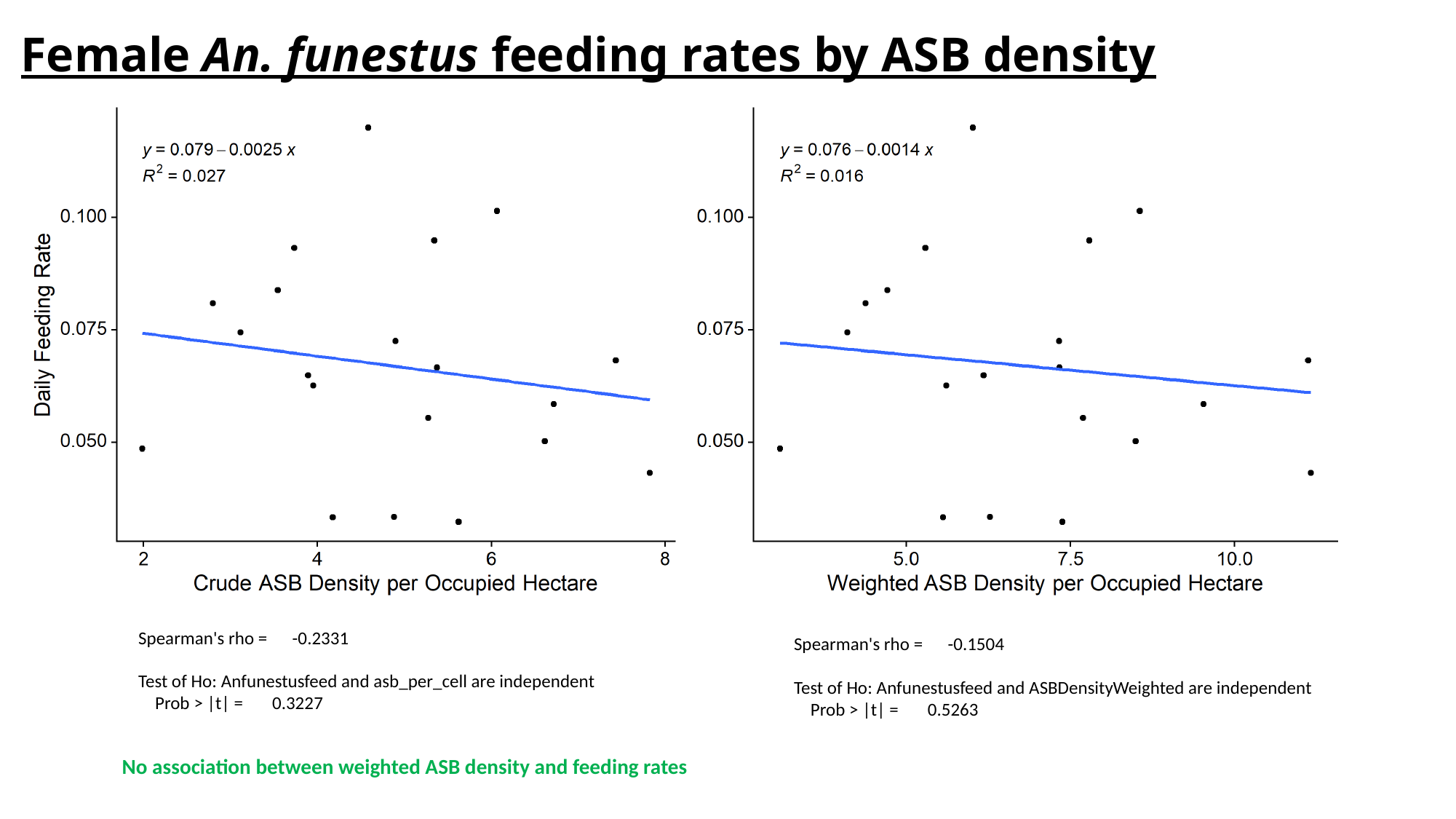

Female An. funestus feeding rates by ASB density
Spearman's rho = -0.2331
Test of Ho: Anfunestusfeed and asb_per_cell are independent
 Prob > |t| = 0.3227
Spearman's rho = -0.1504
Test of Ho: Anfunestusfeed and ASBDensityWeighted are independent
 Prob > |t| = 0.5263
No association between weighted ASB density and feeding rates

## Slide 7
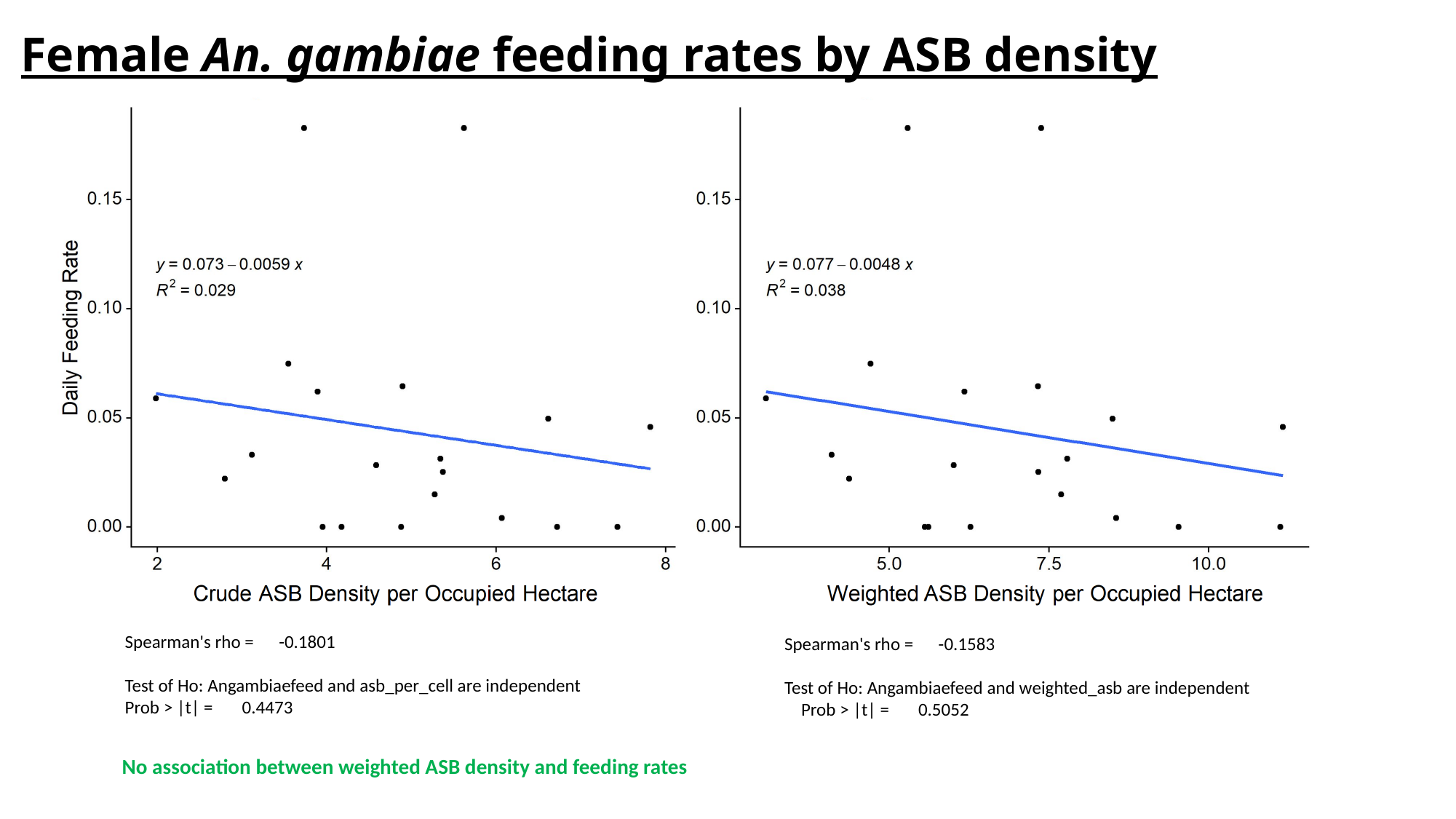

Female An. gambiae feeding rates by ASB density
Spearman's rho = -0.1583
Test of Ho: Angambiaefeed and weighted_asb are independent
 Prob > |t| = 0.5052
Spearman's rho = -0.1801
Test of Ho: Angambiaefeed and asb_per_cell are independent
Prob > |t| = 0.4473
No association between weighted ASB density and feeding rates

## Slide 8
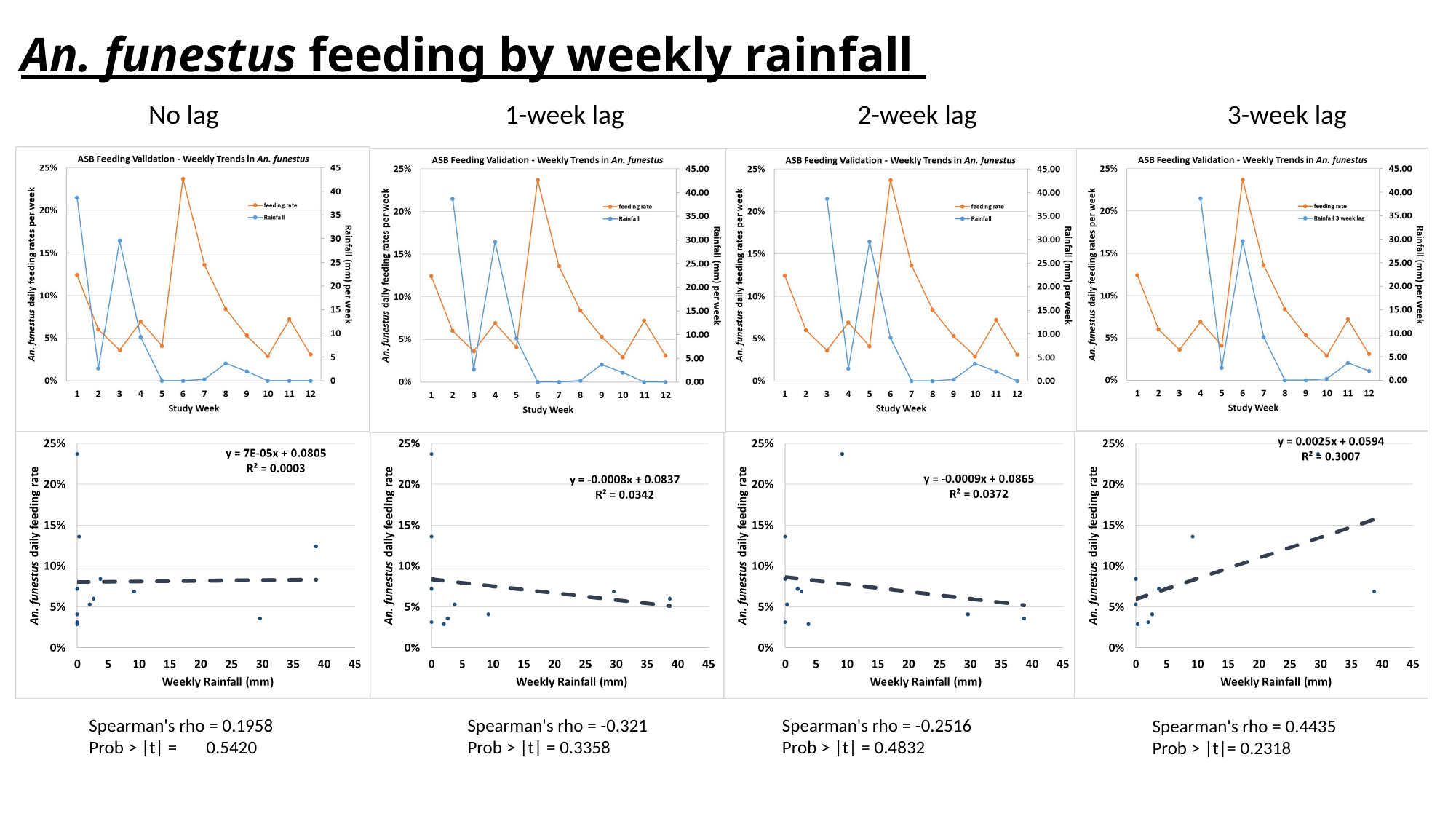

An. funestus feeding by weekly rainfall
No lag
1-week lag
2-week lag
3-week lag
Spearman's rho = 0.1958
Prob > |t| = 0.5420
Spearman's rho = -0.321
Prob > |t| = 0.3358
Spearman's rho = -0.2516
Prob > |t| = 0.4832
Spearman's rho = 0.4435
Prob > |t|= 0.2318

## Slide 9
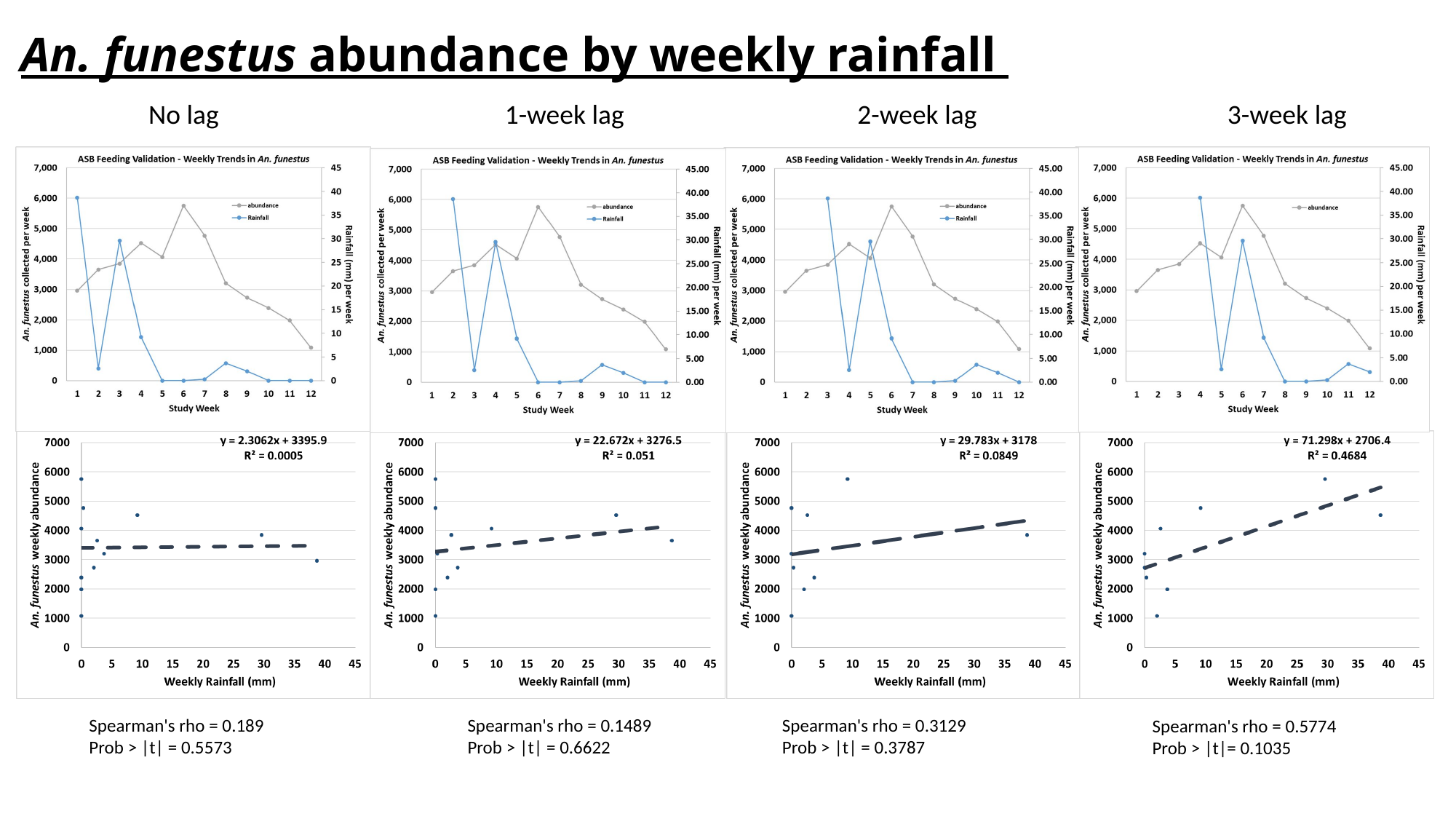

An. funestus abundance by weekly rainfall
No lag
1-week lag
2-week lag
3-week lag
Spearman's rho = 0.189
Prob > |t| = 0.5573
Spearman's rho = 0.1489
Prob > |t| = 0.6622
Spearman's rho = 0.3129
Prob > |t| = 0.3787
Spearman's rho = 0.5774
Prob > |t|= 0.1035

## Slide 10
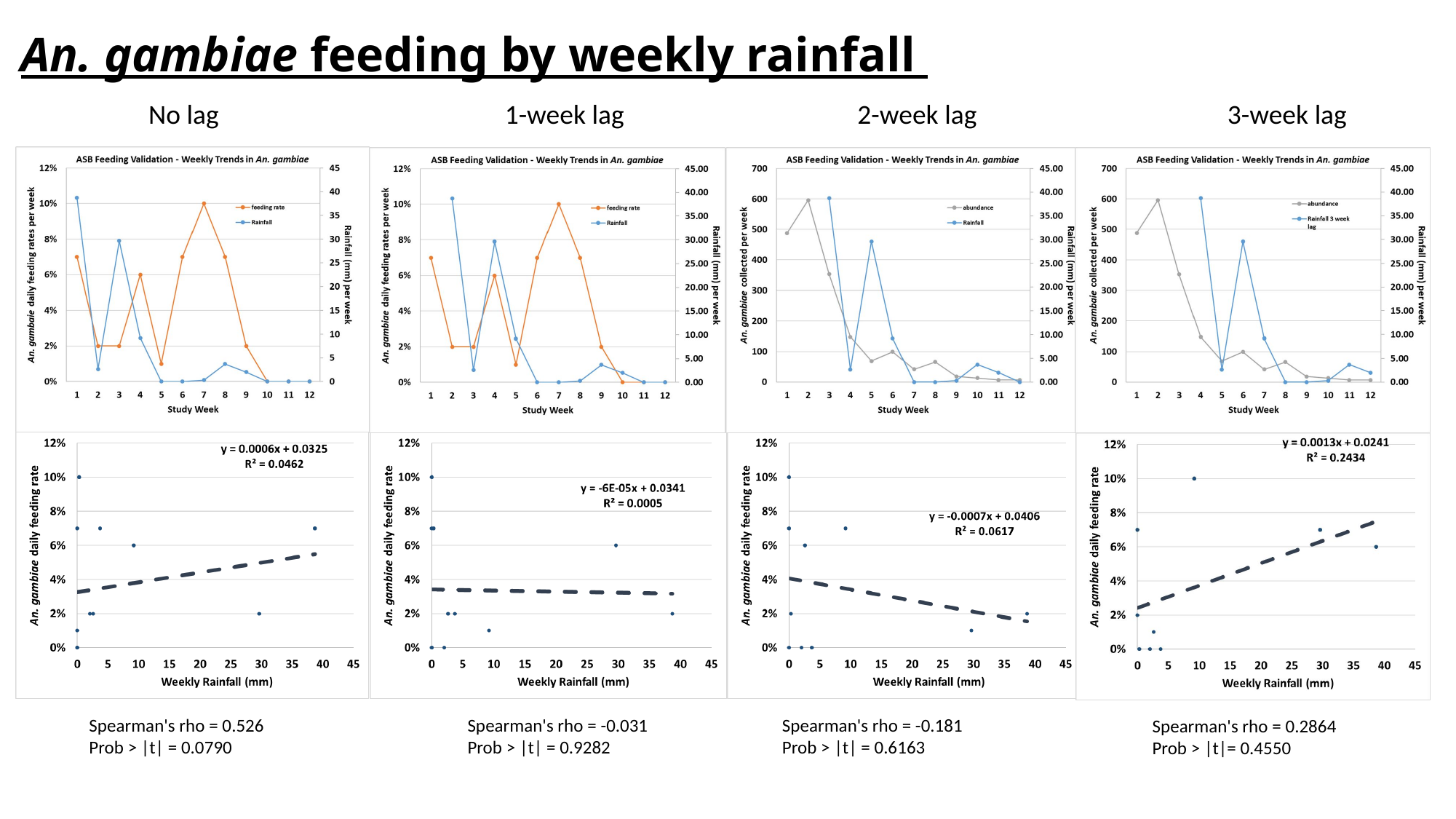

An. gambiae feeding by weekly rainfall
No lag
1-week lag
2-week lag
3-week lag
Spearman's rho = 0.526
Prob > |t| = 0.0790
Spearman's rho = -0.031
Prob > |t| = 0.9282
Spearman's rho = -0.181
Prob > |t| = 0.6163
Spearman's rho = 0.2864
Prob > |t|= 0.4550

## Slide 11
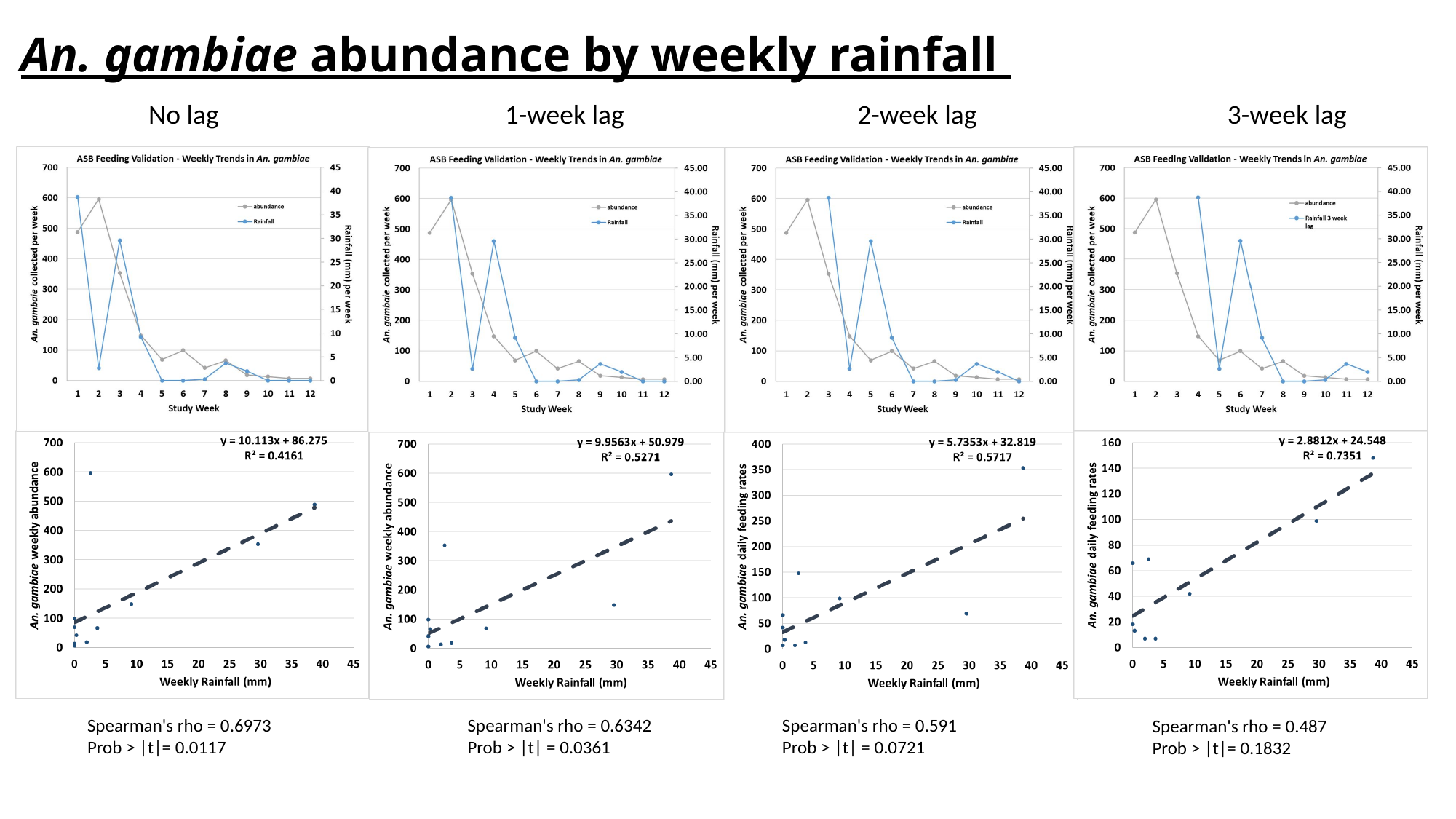

An. gambiae abundance by weekly rainfall
No lag
1-week lag
2-week lag
3-week lag
Spearman's rho = 0.6973
Prob > |t|= 0.0117
Spearman's rho = 0.6342
Prob > |t| = 0.0361
Spearman's rho = 0.591
Prob > |t| = 0.0721
Spearman's rho = 0.487
Prob > |t|= 0.1832

## Slide 12
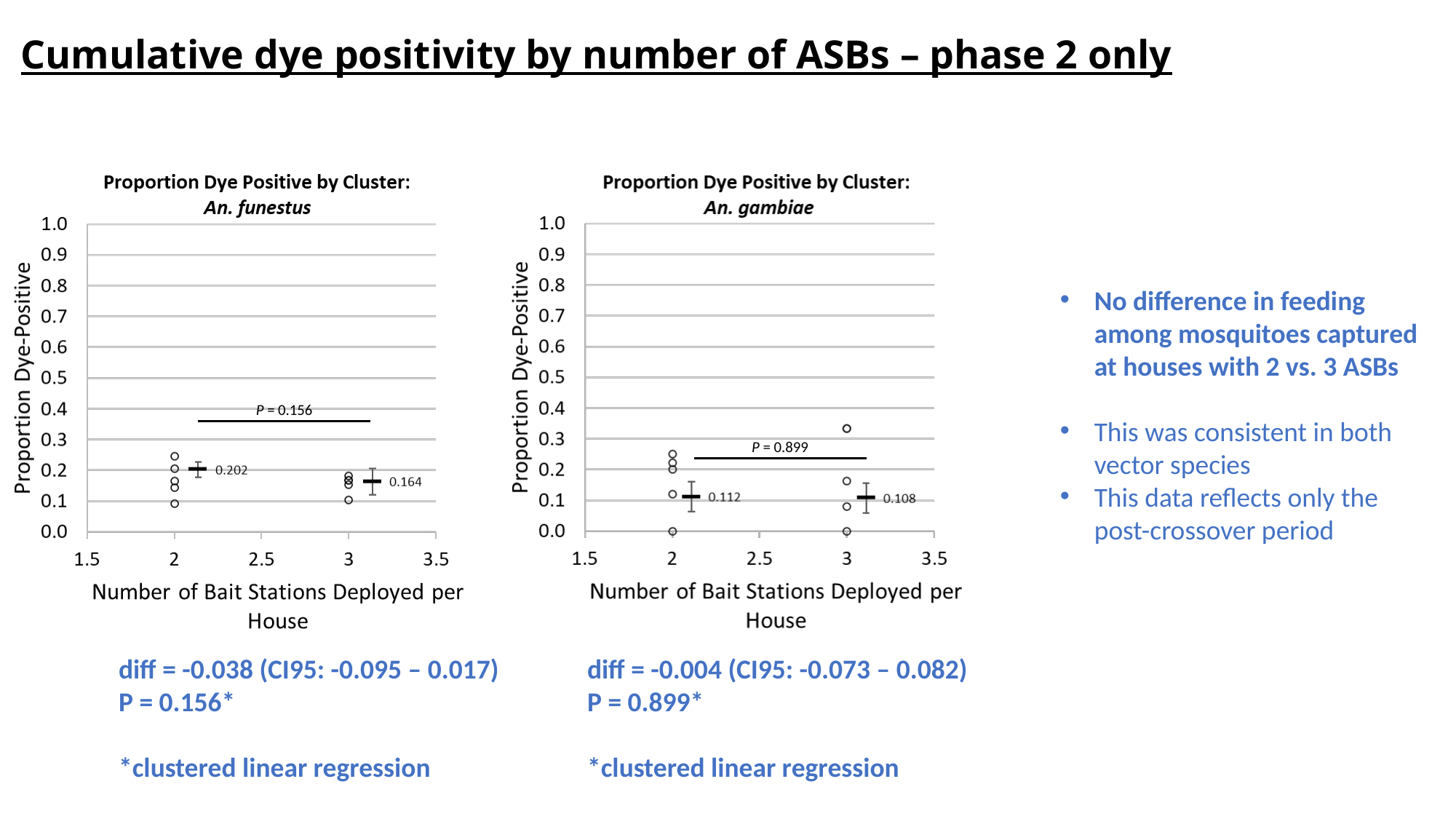

Cumulative dye positivity by number of ASBs – phase 2 only
No difference in feeding among mosquitoes captured at houses with 2 vs. 3 ASBs
This was consistent in both vector species
This data reflects only the post-crossover period
P = 0.156
P = 0.899
diff = -0.004 (CI95: -0.073 – 0.082)
P = 0.899*
*clustered linear regression
diff = -0.038 (CI95: -0.095 – 0.017)
P = 0.156*
*clustered linear regression
